# SATORI: a system for ontology-guided visual exploration of biomedical data repositories

**DOI:** 10.1093/bioinformatics/btx739

**Published:** 2017-11-23

**Authors:** Fritz Lekschas, Nils Gehlenborg

**Affiliations:** 1 Harvard John A. Paulson School of Engineering and Applied Sciences, Cambridge, MA, USA; 2 Department of Biomedical Informatics, Harvard Medical School, Boston, MA, USA

## Abstract

**Motivation:**

The ever-increasing number of biomedical datasets provides tremendous opportunities for re-use but current data repositories provide limited means of exploration apart from text-based search. Ontological metadata annotations provide context by semantically relating datasets. Visualizing this rich network of relationships can improve the explorability of large data repositories and help researchers find datasets of interest.

**Results:**

We developed SATORI—an integrative search and visual exploration interface for the exploration of biomedical data repositories. The design is informed by a requirements analysis through a series of semi-structured interviews. We evaluated the implementation of SATORI in a field study on a real-world data collection. SATORI enables researchers to seamlessly search, browse and semantically query data repositories via two visualizations that are highly interconnected with a powerful search interface.

**Availability and implementation:**

SATORI is an open-source web application, which is freely available at http://satori.refinery-platform.org and integrated into the Refinery Platform.

**Supplementary information:**

Supplementary data are available at *Bioinformatics* online.

## 1 Introduction

Public data repositories are rapidly growing in size and number through implementation of data release policies stipulated by journals and funding agencies (Margolis *et al.*, 2014). The availability of tens of thousands of datasets provides tremendous opportunities for re-use of data across studies. For instance, already published datasets can be used to test a novel hypothesis without having to generate new data. Alternatively, data from previous studies can be employed as corroborating evidence for observations made in an experiment. Meta studies that include data from dozens or hundreds of published datasets are another common use case for the re-purposing of previously generated data. For example, Lukk *et al.* (2010) studied patterns of gene expression in human tissues based on hundreds of public gene expression datasets and a similar study was conducted for mouse tissues by Zheng-Bradley *et al.* (2010). Other groups have studied connections between different diseases using publicly available datasets (Caldas *et al.*, 2009, 2012; Suthram *et al.*, 2010).

To fully embrace sharing and re-use of data, researchers need to be able to (i) find datasets, as described by the *FAIR* (Wilkinson *et al.*, 2016) principles, and (ii) explore data repositories efficiently. As most biomedical raw data are numerical, datasets need to be annotated with metadata to ensure findability. In this context, text-based search is most efficient for navigational queries, e.g. to access a known or recently found dataset, and for some transactional queries, e.g. to find the owner of a known dataset.

But when the exact context of a dataset is unknown or the goal is to learn about the content of a repository, keyword-based search tends to fail (Brandt and Uden, 2003; Holman, 2011) since it provides no overview of the distribution of attributes across the data repository.

**Fig. 1.**
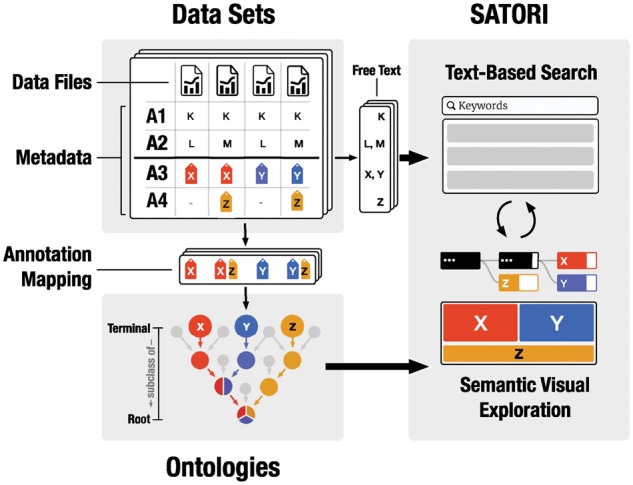
Overview of SATORI’s architecture. Given a collection of datasets, metadata attributes, such as *technology*, *organism* or *disease*, are extracted and indexed as free text for search and ontologically annotated attribute values (e.g. X, Y, Z) are extracted and linked to their related ontology terms. SATORI combines the unstructured metadata and the structured semantic ontology hierarchy to enhance the explorability of datasets through text-based search and visual exploration

Fortunately, an increasing number of data repositories make use of ontologically annotated metadata, which brings context to annotated data attributes. These semantic relationships can be exploited to relate datasets to each other and to provide an overview at different levels of granularity. To address the needs for precise search and exploration, we propose a system that combines free text and ontologically annotated metadata. It consists of two interlinked interfaces: a powerful text-based search and a visual analytics exploration tool (Fig. 1). In the spirit of Pirolli and Card’s (2005) sensemaking process, we aim to enable an improved information foraging (Pirolli and Card, 1995) process by enriching search with attribute-based exploration that visualizes the context of datasets and provides a means of semantic top–down exploration, which is a common approach for exploring unknown data or for analyzing collections (Patterson *et al.*, 2001).

Many biomedical data repositories provide a comprehensive text-based search interface, while a few also support other means of exploration. For example, the two major data repositories for gene expression data are Gene Expression Omnibus (GEO) (Barrett *et al.*, 2013) and ArrayExpress (Kolesnikov *et al.*, 2015), each containing over 70 000 datasets as of May 2017. Besides a text-based search, GEO has an indented list facet view for taxonomy groups and provides a list-based repository browser of high-level features such as sample types or organisms. ArrayExpress has a combined interface for exploring search results and browsing features via list-based filter options, e.g. organisms, experiment type or array. Both of these interfaces are insufficient to fully address the needs of scientists for the exploration of published data. Ontology-guided exploration of data repositories also intersects with search visualization and hierarchical data visualization. For example, InfoSky (Andrews *et al.*, 2002) visualizes hierarchical data collections with circular weighted Voronoi treemaps and ResultMaps (Clarkson *et al.*, 2009) groups search results according to a hierarchical classification visualized using the treemap technique. A detailed evaluation of related work is described in Supplementary Section S1. (Supplementary reference numbers are prefixed with an ‘S’ hereafter.) Still, integrative text-based search and ontology-driven visual exploration have not been fully exploited yet.

In this work, we present a novel, ontology-guided visual analytics tool called SATORI (short for Semantic AnnoTations and Ontological Relations Interface), which combines search and exploration. First, we identified three user roles, their needs and the resulting tasks that should be accomplished by any exploration system (Section 2.1) through a series of semi-structured interviews with experts and analyzed the underlying data structure (Section 2.2). Using this as a starting point, we designed two visualizations that represent the content of a data repository. The visualizations provide users with an overview of the attributes used for annotating the datasets and enable semantic querying (Section 3); implementing the foraging loop of the sensemaking process (Pirolli and Card, 2005). We provide a web-based, open-source implementation of SATORI for the Refinery Platform (http://refinery-platform.org) (Section 3.3).

The Refinery Platform is an end-to-end web application for managing, analyzing and visualizing biomedical datasets. It is build around the Investigation Study Assay (Sansone *et al.*, 2012) data model and relies on a tabular description of datasets. The Refinery Platform provides robust data management and search combined with Galaxy (Afgan *et al.*, 2016) as a powerful analysis back-end. The goal of the Refinery Platform is to integrate the different community standards and therefore needs powerful ways for exploring datasets as a first step in the analysis process.

Using this implementation and two data collections (Stem Cell Commons, Ho Sui *et al.*, 2013 and MetaboLights, Haug *et al.*, 2013), we evaluate our approach in a field study with six bioinformaticians and data curators (Section 3.5).

## 2 Materials and methods

### 2.1 Requirements analysis

We identified three distinct user roles through prior work and user interviews: *data analyst*, *project leader* and *data curator*. While the roles differ in their needs and tasks, they are not mutually exclusive. To guide our design process, we conducted a requirements analysis to determine the needs and tasks for these roles.

**Fig. 2.**
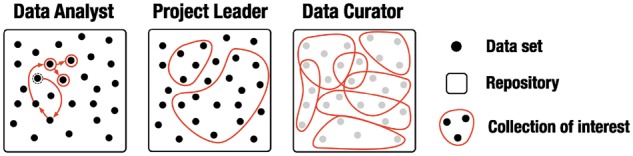
Exploration behavior of different user roles. Data analysts aim at locating specific datasets. Those might be found via *de novo* or iterative search starting from previously known datasets (red arrows). Project leaders focus on collections of datasets and the bigger picture. Data curators are primarily interested in the overall annotation term hierarchy instead of retrieving datasets

The data analyst is mainly interested in finding relevant datasets that help to answer specific biological questions of interest. Their goal is to analyze data and to subsequently transform it into knowledge. The data analyst might start a *de novo* search or continue from an already known dataset to find similar ones (Fig. 2). Therefore, the data analyst needs to *(****N1****) find datasets that match specific experimental attributes* and *(****N2****) find datasets that are similar (or dissimilar) to a given collection of datasets*. The project leader focuses on collections of datasets that are potentially important to accomplish a research project. Their goal is to find multiple datasets (Fig. 2) and generally investigate whether a data resource is useful. Hence, the project leader needs to *(****N3****) get an overview of the distribution of the experimental attributes across a collection of datasets*. Finally, the data curator is interested in the overall state of curation of the entire data repository and is less concerned about retrieving specific datasets. They need to *(****N4****) get an overview of the annotation term hierarchy and term usage*.

Given the user needs, we derived nine tasks by means of semi-structured interviews with eight PhD-level bioinformaticians (Supplementary Section S2.3). The first five tasks are related to learning (Marchionini, 2006) about the content of a data repository. First, the user needs to be able to *(****T1****) find the annotation terms of a dataset*, i.e. to identify attributes of a dataset. To get an idea of the repository’s content the user must also be able to *(****T2****) determine the abundance of annotation terms of a collection of datasets*; e.g. how many datasets are related to *p53*. Furthermore, as the user is often looking for a combination of attributes, it is important to *(****T3****) determine the abundance of multiple annotation terms among a collection of datasets*; e.g. how many datasets are related to *p53*, *liver* and *RNA-Seq*. Since ontologies provide deep hierarchical descriptions of attributes, it is important to enable the user to *(****T4****) understand annotation term containment relationships*. To aid in determining the relevance of datasets, users must be able to *(****T5****) summarize and view the metadata of datasets*. The next four tasks are related to investigating (Marchionini, 2006) data repositories. First, users must be able to *(****T6****) search for datasets*. It should also be possible to *(****T7****) query by annotation terms*. Knowing the annotation term distribution of a given search, it is also important to be able to *(****T8****) loosen annotation term constraints*. For example, when a search for *human RNA-Seq macrophage* returns insufficient results it might be desirable to include all *monocyte*-related datasets to broaden the results. Finally, *(****T9****) ranking annotation terms* is connected to both learning and interacting. Seeing highly abundant terms can help to get an idea of the main data attributes of the repository. On the other hand, annotation terms with a low abundance can highlight the specifics of some datasets.

A detailed description of the relation between user roles, needs and tasks is provided in Supplementary Section S2.

### 2.2 Data

The specifics of data types and structures of biomedical data can vary greatly depending on the research field and application, but the fundamental components for ontology-guided exploration stay the same. As the goal of this work is to find datasets rather than single data files, a dataset is regarded as an atomic unit with multiple attributes associated to the files of a dataset. Some attributes are linked to ontology terms (called *direct annotation terms*). Given the transitive subclass relationships of ontologies, every superclass of a direct annotation term is also associated to the corresponding dataset and denoted as an *indirect annotation term* hereafter. Annotation terms are extracted from the datasets. Hence, the overall number of ontologies and annotation terms depends on the amount to which these datasets have been annotated with ontology terms. More details about the ontology extraction can be found in the Supplementary Section S4.

#### 2.2.1 Abstraction

Ontologies can be considered directed, and in most cases acyclic, graphs in which terms are represented as nodes and relationships as edges between two nodes. For repository exploration, the most important metric of annotation terms is the number of datasets that are associated with the term. Given a graph G=(V,E) with *V* representing the set of vertices and *E* representing the set of edges, we denote the number of times a term *t* has been used to annotate a dataset as the *size* of the term. Terms describe sets of datasets; given a term *t*, its set representation is denoted by *S_t_*. The common root ontology term (i.e. *OWL: Thing*) of ontologies imposes an explicit order on the term sets and defines their term hierarchy. The length of the shortest path of a term *t* to the root term is defined as the distance of *t*. In conclusion, the underlying data structure of ontology-annotated data repositories can be described as a semantic polyhierarchy of attributes, which describe and organize the dataset into groups.

#### 2.2.2 Processing

Most biomedical ontologies describe a specific domain in its entirety but the number of terms that are used for annotation can be very limited. For example, the Stem Cell Commons (Ho Sui *et al.*, 2013) data collection (Section 3.5) uses only 142 out of 1 269 955 ontology terms. Since the goal of SATORI is to provide a means for finding datasets and understanding the composition of data collections rather than visualizing entire ontologies themselves, unused annotation terms are hidden (Fig. 3.1). But even the number of indirectly used terms can be high, given the deep hierarchical structure of some ontologies. Therefore, each parental term should account for a larger collection of datasets than its child term to enable efficient browsing. For example, if a repository contains 10 datasets in total and all datasets are related to *human* then the term *Mammalia* will describe the same 10 datasets as it is an umbrella term that includes human. Hence, the mutual information of all parent terms of *human* related to other attributes (e.g. disease) is zero. Therefore, parental terms of human can be omitted. Thus, the annotation term hierarchy reflects a strict containment set hierarchy. Given three terms *A*, *B* and *C* where *A* is a subclass of *B* and *B* is a subclass of *C*, the set representations *S_A_*, *S_B_* and *S_C_* of the terms should fulfill:

(1) $$S_{A}\subset S_{B}\subset S_{C}\;$$

This leads to pruning of terms whose size is zero as illustrated in Figure 3.2. For example, Stem Cell Commons (Ho Sui *et al.*, 2013) includes datasets with files sampled from three species: *human*, *mouse* and *zebrafish*, which have all been annotated with NCBITaxon (Federhen, 2012). Pruning the subgraph starting from the last common ancestor (*euteleostomi*) according to Equation (1) results in the removal of 37 terms (Supplementary Fig. S3).

**Fig. 3.**
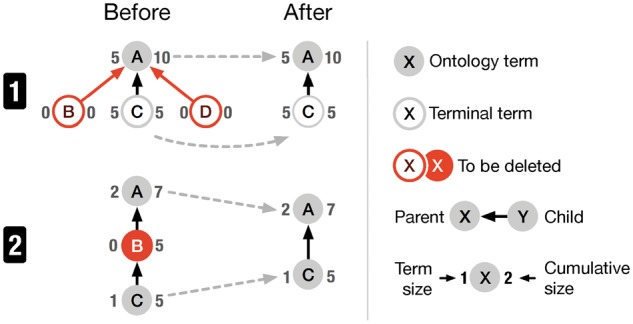
Annotation term pruning: to reduce the size of the annotation term graph and make exploration more efficient, terminal (1) and inner (2) ontology terms of size zero (i.e. terms that have not been used for annotation) are not displayed (red nodes)

## 3 Results

The key goal for SATORI is to enable researchers to more efficiently re-use existing dataset by improving the explorability of data repositories through a combination of traditional free-text-based search and context-describing ontology annotations of the metadata.

### 3.1 Interface and visualizations

**Fig. 4.**
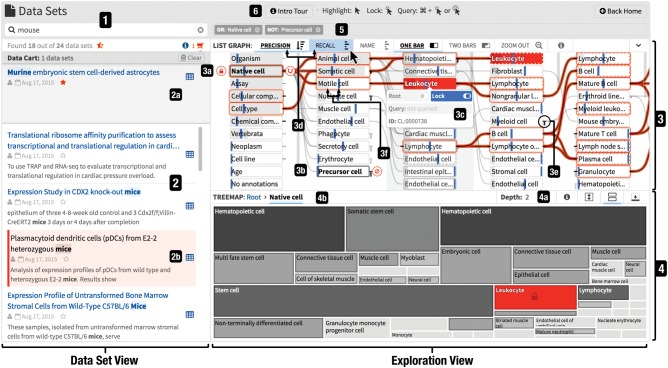
The interface of SATORI consists of the dataset and exploration view. The dataset view contains the search interface (1) and the list of retrieved datasets (2). Each dataset is represented with a surrogate (2b) and can be saved in the data cart (2a). The exploration view is composed of the node-link diagram (3), treemap (4) and query term bar (5). SATORI features several interactive tutorials, indicated by info buttons (6). This example illustrates a query for ‘native cells’ (3a, 4b) excluding ‘precursor cells’ (3b) combined with a synonym keyword search for ‘mouse’(1); i.e. the retrieved datasets contain the word ‘mouse’ (or synonyms like ‘mice’) in their free-text description, have been annotated with ‘native cell’, and do not contain ‘precursor cell’ annotations. Among these retrieved datasets, those annotated with ‘leukocyte’ are highlighted (2b) via the node context menu (3c). The recall, highlighted as blue vertical bars (3d), of leukocyte is less than 50% and the depth of the treemap is set to 2 (4a) to provide a broader overview

SATORI is composed of three interlinked views: dataset view, exploration view and dataset summary view. The first two components are visible by default as shown in Figure 4. The dataset summary view is displayed on demand (Supplementary Fig. S9) only.

#### 3.1.1 Dataset view

The dataset view is composed of a text-based search interface (T6), a list of datasets and a data cart. The design of the search interface has been kept at a minimum to be easy to use (Nielsen, 1994). A dataset is represented by a surrogate—a short description consisting of the title, ownership and sharing information, and an indicator whether the dataset is currently saved in the data cart. Additionally, search results feature a *keyword in context* snippet to provide context to the matched keywords (Fig. 4.2b and Supplementary Fig. S4). A click on the title of a dataset opens the dataset summary view. Being able to quickly get an overview of the metadata of a dataset is crucial for evaluating the relevance of the dataset in regard to the information need (T5).

The data cart (Fig. 4.2a and Supplementary Fig. S5) integrates into the dataset view and enables users to temporarily collect datasets of interest during the exploration process. This reduces the cognitive load during search when comparing results from different searches or annotation term queries as users do not need to memorize the description of datasets.

#### 3.1.2 Exploration view

The exploration view contains two visualizations showing the content of a data repository in terms of the metadata attributes: a node-link diagram (Fig. 4.3) and a treemap plot (Fig. 4.4). Both display the same data but represent attributes differently to compensate for each other’s limitations. The treemap provides a space-efficient overview of higher-level terms and the node-link diagram represents the relationships between terms across multiple levels.

**Fig. 5.**
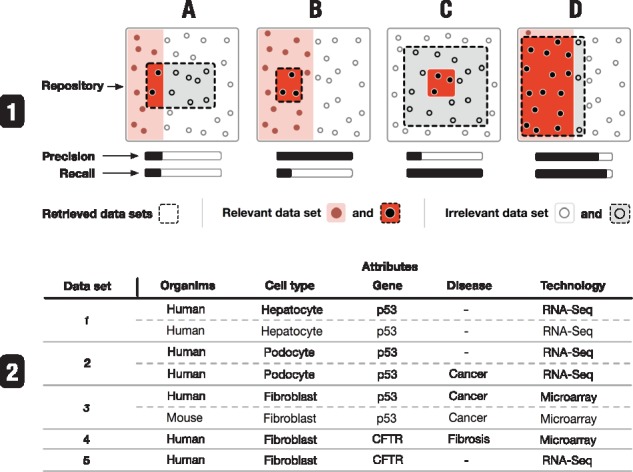
Four cases illustrating the utility of precision and recall. (1**A**) indicates that an annotation term (i.e. attribute) is not directly related to the search. For example, given (2), in a broad search for ‘human’, ‘fibrosis’ is not frequently associated with the datasets found. (1**B**) states that more datasets related to the annotation term are available. For instance, a search for ‘human hepatocyte p53’ will result in a low recall for *p53* as datasets 2 and 3 are not retrieved since they are not associated with hepatocyte. (1**C**) illustrates an annotation term that describes a subgroup of all retrieved datasets. For example, a search for ‘human fibroblast’ shows high recall but low precision for ‘fibrosis’, indicating that it is not a commonly studied disease among the retrieved datasets. Finally, (1**D**) indicates an annotation term that describes many of the retrieved datasets, e.g. a search query for ‘RNA-Seq’ leads to a high recall and precision for p53

In the treemap, each term is visualized as a rectangle. The area of the rectangle represents the size of the term relative to its sibling terms. The color indicates the distance to the farthest child term, i.e. the subtree’s depth. The farther away a child term is, the darker is the color of the rectangle. The node-link diagram visualizes terms as nodes and links parent and child terms according to subclass relationships defined by the ontologies. Additionally, the node-link diagram shows the precision and recall for each term given the currently retrieved datasets. Precision is defined as the number of datasets annotated with a term divided by the total number of retrieved datasets. Recall is defined as the number of retrieved datasets annotated with a term divided by the total number of datasets annotated with this term across the entire repository. Precision is useful to understand how frequently a term is used for annotation in the retrieved collection of datasets while recall provides a notion of information scent (Pirolli *et al.*, 2000) as it specifies how many relevant datasets for a specific attribute have been retrieved in total (Fig. 5).

#### 3.1.3 Treemap

The treemap technique visualizes the size of annotation terms (i.e. the number of datasets annotated with a certain term). A main advantage is that the currently selected tree level is always drawn without any overflow or occlusion, which provides an immediate overview. Other visualization techniques that are used for deep hierarchies, e.g. indented lists or node-link diagrams, typically require user interactions to uncover hidden parts. This forces the user to memorize hidden parts. Therefore, the main focus of the treemap is on aggregation of the content by annotation terms. On the other hand, it is hard to perceive the hierarchical structure with treemaps (van Ham and van Wijk, 2003). Also, the rectangle areas are not relative to the root and engender ambiguities (Supplementary Fig. S12). Finally, the area encoding is relatively imprecise compared to other encodings like length (Heer and Bostock, 2010). The node-link diagram compensates for these disadvantages. The treemap also features a breadcrumb path that shows the parental annotation terms to the absolute root term (Fig. 4.4b and Supplementary Fig. S8.1) to support T4 and T8. Additionally, the visible depth of the treemap can be increased, via the distance control of the treemap (Fig. 4.4a and Supplementary Fig. S8.2). Showing multiple levels of the hierarchy provides better understanding of the structure at the cost of readability.

#### 3.1.4 Node-link diagram

The node-link technique provides a strong visual notion of connectedness between related annotation terms and emphasizes the hierarchical structure of the annotation term graph (T4). Two terms are visually linked when they are related by an ontological ‘is superclass of’ relationship. The directionality follows the reading direction from left to right. To avoid visual clutter, edges are only drawn if both nodes are visible. To indicate the number and position of omitted links, a bar is displayed left or right of a term for incoming and outgoing links to nodes outside the visible area (Fig. 4.3e and Supplementary Fig. S10). Annotation terms are ordered in individually sortable (T9) and scrollable columns by their distance to the root term and aligned to the top to increase the overall space efficiency (Supplementary Fig. S2). The horizontal layout has been chosen over a vertical layout to make paths follow the reading direction, to easily compare *precision* and *recall* (T2 and T3) within a column via aligned bars and to provide familiar scroll behavior (i.e. top–down).

By default, the superimposed bar in nodes displays precision and the superimposed vertical line indicates recall. The visual representation for precision and recall depends on which attribute the nodes are ordered by. As users typically start exploring the entire repository, recall is initially equal to 1 and thus less informative than precision. Therefore, by default nodes are sorted by precision. The top navigation bar allows to sort nodes, adjust the bar style and zoom-out to see the entire graph. In Figure 4.3d nodes are ordered by precision, which is visualized as superimposed gray bars. Recall is highlighted as superimposed blue vertical lines by placing the mouse cursor over the recall button. For example, *motile cell* (Fig. 4.3f) has a precision of about 0.2 and a recall of about 0.5.

#### 3.1.5 Dataset summary

The dataset summary view supports the *reading* and *information exporting* step of the information foraging loop (Pirolli and Card, 2005) and addresses T5 (i.e. ‘summarize and view the metadata of a dataset’). The layout has been designed to reflect the importance of attributes of datasets for exploration, which we derived from the initial semi-structured interviews (Supplementary Table S2 and S3).

### 3.2 Interactions and querying

All components of SATORI are linked to provide an integrative exploration experience and to visualize the semantic context of retrieved datasets.

#### 3.2.1 Identify dataset-associated annotations

When hovering over a dataset surrogate all associated annotation terms are highlighted in the treemap and node-link diagram (T1). Since the node-link diagram shows nodes across different hierarchical levels, direct and indirect annotation terms are handled differently: direct annotation terms are filled in orange while indirect annotation terms only feature an orange outline (Fig. 4.3). The node-link diagram can exceed the visible area in size, hence some parts might be occluded. To focus on the specific annotation terms of a mouse-hovered dataset only, the user can semantically zoom out via a click on the magnifier button such that all terms related to the mouse-hovered dataset become visible (Supplementary Figs S4 and S11).

#### 3.2.2 Discover datasets by annotations

To explore the content of a repository based on annotated attribute values, SATORI highlights associated dataset surrogates (Fig. 4.2b and Supplementary Fig. S6) when moving the mouse cursor over a term in either of the two visualizations (T2). To investigate annotations across views, a click on a rectangle in the treemap or the *Lock* button of the context menu (Fig. 4.3c and Supplementary Fig. S7) of the node-link diagram will make the highlighting persistent. Both visualizations support term-based querying to address T7, T8 and T9. A double click on a rectangle in the treemap will zoom into the subtree and simultaneously restrict the retrieved datasets to be associated with the subtree’s root term, hence the dataset collection is queried for the clicked term (T7 and T8). The same action can be triggered in the node-link diagram via a click on the *Root* button in the term context menu (Fig. 4.3c and Supplementary Fig. S7). Loosening annotation constraints (T9) can be achieved through a click on the treemap’s breadcrumb-like root path view (Fig. 4.4b and Supplementary Fig. S8) or by deactivating rooting via another click on the *Root* button in the term context menu. Additionally, the node-link diagram supports more complex Boolean queries for annotation terms with context menu’s *Query* button (T3 and T7). Four query states are implemented: *none*, *or*, *and* and *not*, which the user can toggle through by clicking multiple times on the Query button. Since queries alter the visual state of the visualizations it can get complicated to remember executed queries. Therefore, the query term interface displays all query terms and supports removing queries or altering query states (Fig. 4).

### 3.3 Implementation and scalability

SATORI is a web-based exploration system. The front-end is implemented in JavaScript using D3.js (Bostock *et al.*, 2011) and AngularJS. The information retrieval system is powered by Solr and ontologies are stored in a Neo4J graph database. While Solr provides access to the metadata, Neo4J stores the complete ontology graph. A custom Java plug-in provides access to and retrieves the user-specific annotation subgraph for visualization. The Refinery Platform application manages the dataset collection and controls the business logic between Solr and Neo4J. Supplementary Figure S13 shows an overview of SATORI ’s architecture. All parts of SATORI are open-source and publicly accessible at GitHub (https://github.com/parklab/refinery-platform) (https://github.com/flekschas/d3-list-graph) and continuously integrated via Travis-CI to ensure correctness and compatibility.

#### 3.3.1 Ontology representation in Neo4J

We have developed a simplified property graph representation for ontologies to provide space-efficient storage and fast access. Each class is stored as a node with a few core properties like the Uniform Resource Identifier and label. Superclasses are by default related to the class via a ‘subclass of’ relationship. Other relationships, such as *existential quantification properties*, can be incorporated by adjusting our converter (https://github.com/flekschas/owl2neo4j), which translates OWL-formatted ontologies into the property graph model used by Neo4J. To save space when working with multiple ontologies and to speed up node retrieval, every class corresponds to one node, which is labeled with a user-defined ontology abbreviation, e.g. EFO for the Experimental Factor Ontology (Malone *et al.*, 2010). This enables us to traverse a single graph but still trace the occurrence of each class (Supplementary Section S4).

Since biomedical data repositories can grow quickly, scalability is an important property of any repository exploration system. The performance of SATORI foremost depends on the total number of distinct annotation terms. The impact of the number and size of datasets or ontologies used is negligible since Solr and Neo4J are capable of handling millions of documents and only classes that are directly or indirectly used for annotation are retrieved. Currently, the node-link diagram is the limiting factor as it displays the full annotation graph. We have tested the tool with up to 1000 annotation terms and while the performance decreases, the tool remains usable.

### 3.4 Usage scenario

In this section, we illustrate three usage scenarios for SATORI using the Stem Cell Commons (Ho Sui *et al.*, 2013) and MetaboLights (Haug *et al.*, 2013) data collections, which are comprised of 119 public and a selection of 200 datasets respectively. Datasets of Stem Cell Commons are annotated with 12 ontologies (Supplementary Table S1) and datasets of MetaboLights are annotated with more than 40 ontologies (Supplementary Table S2). The Supplementary Video (https://youtu.be/WpbBoW2f4iM) illustrates the following usage scenarios in detail.

#### 3.4.1 Context-driven search

Assume a data analyst is searching for datasets related to leukocytes in Stem Cell Commons. A search query using this term does not retrieve any datasets as no datasets have been explicitly annotated with leukocytes. To evaluate whether similar datasets are available, the data analyst browses the repository by cell types. To restrict the datasets and annotation graph to a specific cell type, the data analyst selects *native cell* as a root term (Fig. 6.1A). Three prominent subgroups (*somatic cells*, *animal cells* and *precursor cells*) become apparent. As leukocytes are not precursor cells the data analyst chooses to filter out datasets related to precursor cells (Fig. 6.1B). The next column of child terms contains *leukocyte*, so the data analyst performs an *and* query with it, which retrieves five datasets (Fig. 6.1C). Ordering nodes by recall shows that less than 50% of all datasets related to *leukocyte* have been retrieved (Fig. 6.1D). The data analyst removes the query terms via the query term bar (Fig. 4.5) and retrieves all 12 datasets associated to *leukocytes*. Querying by *leukocyte* and *organism* reveals that the retrieved datasets are associated with *mouse* (Fig. 6.1E). Using the dataset preview, the analyst finds out that the datasets are related to subtypes of *leukocyte*, which is why the search did not retrieve any datasets.

**Fig. 6.**
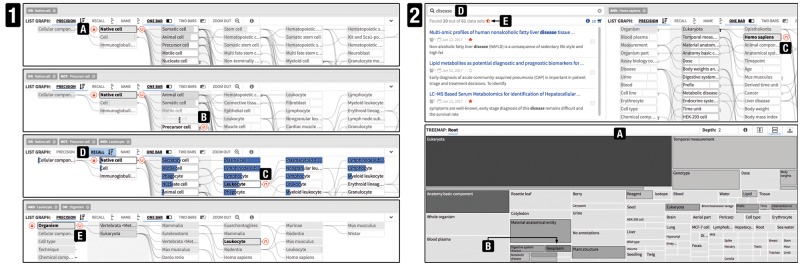
Illustrating steps in two scenarios described in Section 3.4. (1) Four steps in retrieving leukocyte-related datasets, utilizing all four query modes (**A**, **B** and **C**), recall (**D**) and inter-branch querying (**E**). (2) Diverse annotation structure of MetaboLights (**A**) and the combination of querying and searching (**C** and **D**)

#### 3.4.2 State of data curation

Assume a data curator wants to evaluate the curation state of Stem Cell Commons. The data curator maximizes the treemap visualizations to get an overview of the distribution of the annotation terms. First, the data curator increases the visible depth of the treemap to identify large terminal terms, i.e. rectangles without a border, that represent classes with no subclasses. Such terms could potentially be described in more detail. At depth two, *biotin* pops up as a large terminal node. Zooming into *biotin* reveals that 69 out of 119 public Stem Cell Commons datasets are annotated with it, which is not surprising given that many datasets contain microarray or RNA-Seq experiments. Zooming out to the parent chemical component reveals that only one other term, representing a *chemical component*, is used for annotation, which leaves room for future improvement. Investigating *assay*, another term with two large terminal child terms, reveals that only 116 out of 119 are annotated with *assay*. Using the *not* query mode, the data curator figures out that the three datasets missing the *assay* annotation are actually related to it as well, in particular *epigenetic modification assay*; highlighting another area for improvement.

#### 3.4.3 State of data availability

Assume a project leader wants to learn about the content of MetaboLights, in particular diseases-related studies. A first look at the treemap shows that MetaboLights contains a high diversity of studies as represented by many small terminal nodes (Fig. 6.2A). The term *neoplasm* pops out given it relative dark background (Fig. 6.2B), indicating more specific subtypes. Zooming into *neoplasm* reveals three datasets, which the project leader stores in the data cart for future comparison. The low recall of *disease*, the parent term of *neoplasm*, indicates that more disease-related datasets are available. Zooming out and querying for disease using the node-link diagram enables the project leader to check which species have been studied in the context of the disease-related datasets. *Rooting* the node-link diagram for *eukaryotes* reveals that most disease-related studies are associated with *human* and some with *mouse*. Having queried (Fig. 6.2C) and saved the 10 *human*-related datasets in the data cart, the project leader clears the annotation query and instead searches for *disease* to find 20 datasets (Fig. 6.2D). Some, but not all, of the datasets overlap with the previously saved datasets (Fig. 6.2E). The project leader saves the remaining datasets as well for future project discussions.

### 3.5 Evaluation

We conducted a field study with six bioinformaticians and data curators on the Stem Cell Commons data collection (Section 3.4) to evaluate the general utility of SATORI in exploring a biomedical data repository. Five of the participants are PhD-level scientists and one is a graduate student. The study consisted of a brief introduction to SATORI, a set of *kick-off* tasks for browsing of the Stem Cell Commons data collection (Supplementary Table S5) and open-ended exploration. During the 1 h long exploration we recorded anecdotal evidence about SATORI. The participants consist of four data analysts, one project leader and one data curator to cover all identified user roles (Section 2.1). Three of the six participants were recurring participants from the initial interviews (Supplementary Section S2.3).

All of the participants stated that SATORI gives them a better understanding of the content of the entire repository compared to a system with only text-based search. Two data analysts mentioned that the ontology-guided exploration interface is very useful for collecting datasets associated with higher-level attribute values, which are not mentioned in the dataset description (e.g. *neoplasm* as compared to *glioma*). The project leader mentioned that SATORI significantly aids exploration of unknown big data collections. The data curator said that ‘it is really exciting to finally see and explore the (curated meta)data’ and that it will be a useful asset for future data curation.

A drawback, identified by all participants, is the difficulty to locate specific terms within the two visualizations. They would like to be able to search for annotation terms. Also, all participants mentioned at the end of the session that SATORI requires some training or introduction. In response, we implemented several step-by-step guides to help first-time users. Also, everyone agreed that many high-level annotation terms are too generic and not useful for exploring data repositories as they are associated with almost all datasets. We have addressed this by defining a set of more useful terms (Supplementary Table S6 and S7) as entry points. Finally, participants stated that it can currently be time consuming to figure out the current state of querying. We added the query term bar (Fig. 4.5), which displays all active queries, to address this concern.

## 4 Discussion

The feedback collected in our field study (Section 3.5) provides evidence that SATORI addresses the needs of the three-defined user roles (Section 2.1) and supports the tasks (Section 2.1) in exploring data repositories. Although SATORI is currently integrated into the Refinery Platform, it can be adapted to any data repository that uses controlled and hierarchically organized vocabularies for annotation. For example, data repositories managed by the European Bioinformatics Institute like ArrayExpress (Kolesnikov *et al.*, 2015) or MetaboLights (Haug *et al.*, 2013) already have ontologically annotated datasets and could easily integrate SATORI into their systems.

A common challenge in visualizing ontology-driven set hierarchies is that many bio-ontologies define complex polyhierarchies with different levels of class granularity. A trade-off has to be made between complexity and usability. Pruning the ontology graph to represent a strict containment hierarchy is a first step but there are more opportunities to improve which class of a pruned branch is kept and to better support visual representation of polyhierarchies. Also, SATORI focuses on exploration of data repositories by finding datasets based on metadata attributes rather than comparing groups of metadata attributes. The ability to compare different groups of annotation terms is currently limited. Existing visualization techniques for exploring set intersections, such as UpSet (Lex *et al.*, 2014), could be integrated into SATORI to enable richer comprehension of term-related set properties.

## 5 Conclusions

SATORI is a web-based exploration system that combines powerful search with visual browsing to provide an integrated exploration experience. The visualizations serve two purposes: supporting the information foraging loop (Pirolli *et al.*, 2000) and pattern discovery of attribute distributions, as well as ontology-guided semantic querying of the data repository. SATORI contributes to the biomedical domain by unifying text-based search with visual exploration approaches, which put datasets into context and shed light in the repository-wide distribution of biological attributes. SATORI extends upon findability of datasets, as described in the FAIR (Wilkinson *et al.*, 2016) principles, by enhancing explorability of data repositories, which is a crucial property as data repositories keep growing at a continuous rate. It is clear that—apart from the design of the visualization and the implementation—the greatest challenge of any semantic exploration approach is that it significantly depends on the quality of data curation. Inconsistent or lacking ontology annotations can result in significant discrepancies between the free-text search and annotation term queries. SATORI also enables curators to evaluate the current state of curation and identify areas that need improvements. We also observed that, due to the nature of the complexities of ontologies, ontology-guided exploration tools require initial learning and are currently most useful for expert users. SATORI focuses on exploration of data repositories given a fixed annotation state. Tracing changes in this annotation space due to ongoing data curation or updated ontologies is an important and unsolved challenge, which requires research on ontology versioning and semantic comparisons.

### 5.1 Future work

To better support locating annotation terms we propose a unified visual query interface, which handles text-based free text search, annotation term search, annotation term query operations and basic filtering. This would simplify locating specific annotation terms and could encourage more people to explore semantic annotations. While the user study presented in this paper indicates that SATORI is useful for different user types in exploring data repositories, long-term quantitative user studies are needed to evaluate how analysts interact with the system in day-to-day use. Finally, integrating non-ontologically structured metadata into SATORI could have a notable impact as not all descriptive metadata is ontologically annotated.

## Supplementary Material

Supplementary Data
