# Supplementary material for: SATORI: a system for ontology-guided visual exploration of biomedical data repositories
Source: Bioinformatics. 2017 Nov 23;34(7):1200–7. doi: 10.1093/bioinformatics/btx739 (PMC6031061; doi:10.1093/bioinformatics/btx739)
Supplement: Supplementary Data [file btx739_supp.zip › btx739-suppl_data/SATORI_supplementary_material.pdf]

# SATORI: A System for Ontology-Guided Visual Exploration of Biomedical Data Repositories

## Supplementary Material

Fritz Lekschas
lekschas@seas.harvard.edu
Harvard John A. Paulson School of Engineering and Applied Sciences
Harvard Medical School

Nils Gehlenborg
nils@hms.harvard.edu
Harvard Medical School

Oxford Bioinformatics, 2017

# Supplementary Information

## 1 Related Work

Ontology-guided exploration of biomedical data repositories intersects with a number of different research areas. To provide a comprehensive overview of related work, we've reviewed two main areas: search visualization as well as tree and graph visualization methods.

### 1.1 Methods for Search Visualization

Exploration of data repositories or large document collections have similar requirements and goals as search visualization, since search results represent an arbitrary subset of the document corpus. Additionally, search visualization incorporates the notion of relevance for retrieved data sets.

Over the last two decades, various visualization methods have been developed to support search. The tools can be divided into those that attempt to visualize each result separately and those that try to provide an overview of the complete result space. For example, TileBars (Hearst, 1995) and its successors Insyder (Reiterer *et al.*, 2005), and HotMap (Hoeber and Xue Dong Yang, 2006) visualize the approximated location of query term matches within each retrieved document and thus provide a visual notion of relevance. Others illustrate the relative similarity of search results by depicting each retrieved document as a glyph or a simple visual mark in a 2D or 3D space, where the spatial location is mostly determined via dimensionality reduction. Similar documents should cluster together and form fuzzy groups or categories. Examples for glyph-based visualization techniques that operate on search results are InfoSky (Andrews *et al.*, 2002) and xFind's VisIslands (Andrews *et al.*, 2001). InfoSky incorporates hierarchical classification of the documents and displays them in circular weighted Voronoi treemaps.

Other visualization methods provide an abstract summary of the set of all retrieved documents. The RelationBrowser++ (Zhang and Marchionini, 2004) visualizes the overall and search related abundance of categories using superimposed bar charts on category labels. The search engine Grokker categorizes search results hierarchically and provides a top-down filter mechanism via a circular treemap of topics and subtopics. Note that Grokker was shut down when Groxis ceased operations in 2009. The Internet Archive provides a copy of Grokker's tour, which contains screenshots and a brief explanation of their visualization tool (`Web Archive`). ResultMaps (Clarkson *et al.*, 2009) groups search results according to a hierarchical classification and uses the treemap visualization technique to convey the hierarchy. Hearst provides a comprehensive overview of the efforts in visualizing search results (Hearst, 2009).

Apart from that, a number of projects have studied possibilities to visually summarize the corpus of data repositories and enable exploration. The following examples focus on visual exploratory tools that utilize metadata or descriptive vocabulary, i.e., tools that visualize categorized or set-typed data. InfoSky (Andrews *et al.*, 2002) can also be used to explore data collections as a whole. Hiérarchie (Smith *et al.*, 2014) is a tool for visualizing hierarchical topic models using sunburst charts for exploration of text documents. In a similar fashion, PhenoBlocks (Glueck *et al.*, 2016) uses the SunBurst idiom to represent the hierarchical structure of the Human Phenotype Ontology (Robinson *et al.*, 2008).

Even though SATORI only visualizes a very limited subset of the complete ontologies, it is worth mentioning that many efforts went into visualizing ontologies in their entirety. Katifori *et al.* (2007) provide an extensive overview of different visualization techniques. Most methods focus on a representation for certain details, e.g., indented lists show the sub- / superclass relationships. VOWL (Lohmann *et al.*, 2014), on the other hand, is a specification for visualizing the whole structure of an ontology using the node-link idiom and predefined visual marks for different aspects of an OWL ontology.

### 1.2 Methods for Tree- and Graph Visualization

Other work that indirectly relates to SATORI is more focused on visualization techniques for graph, tree, or hierarchical containment data. The variety of tree visualizations alone is huge. Schulz (2011) maintains an extensive collection of numerous different visualization methods for tree data. Treemaps (Johnson and Shneiderman, 1991) are one of the most space efficient ways to visualize hierarchical data and have been studied extensively. A major disadvantage of treemaps is that they do not communicate the tree topology as well as node-link diagrams do. Elastic Hierarchies (Zhao *et al.*, 2005) has been developed to combine the strength of node-link diagrams and treemaps. GrouseFlocks (Archambault *et al.*, 2008) is another attempt to combine the node-link idiom with circular treemaps. Jigsaw's (Stasko *et al.*, 2007) list view illustrates relatedness of different items in lists via color coding and linking of query-related items. Parallel tag clouds (Collins *et al.*, 2009) arrange feature words of different text corpora in parallel lists and highlight identical feature words via indicated links that get fully drawn once the user interacts with the visualization.

## 2 Roles, Needs, and Tasks

### 2.1 User Roles

Both authors have previously worked on data repositories and are familiar with biomedical informatics research. Therefore we are aware that data repositories are used by different types of users or that users can take different roles, which we characterized prior to undertaking our task analysis. We identified the following three primary user roles in the context of exploring biomedical data repositories:

**R1** data analyst

**R2** project leader

**R3** data curator

These user roles are not mutually exclusive. For example, a single person can act as a *data analyst* and *project leader* at the same time.

### 2.2 Needs

The primary concern of *data analysts* is turning experimental data into information and subsequently transforming this information into knowledge by answering questions of interest. Given biomedical data, a *data analyst* may be searching for data sets that are most relevant to a given biological problem or question. A precise description of the experimental attributes is most important to assess relevance. While the attributes that matter most can vary greatly depending on the project, the goals for finding relevant data sets are often similar such as finding data to test the validity of a hypothesis, complementing in-house generated data to improve confidence, to compare quality between different data sets, to check the robustness of an algorithm, or to broaden the scope of a study. In order to achieve these goals the *data analyst* needs to:

**N1** find data sets that match specific experimental attributes.

**N2** find data sets that are similar (or dissimilar) to a given collection of data sets.

The *project leader* is somebody who leads a group of data analysts and is mainly focused on finding collections of data sets. This could be a professor trying to plan a new study or a grant proposal. Here the foremost goal is to ensure that the group has access to data that will allow them to address new challenges in

their scientific area. To achieve this, *project leaders* need to be able to find out whether a repository contains data sets matching certain experimental attributes of interest, to get an overview of the current state of the repository, and to discover trends in data generation and availability. Therefore, the primary need of a *project leader* is to:

**N3** get an overview of the distribution of the experimental attributes across a collection of data sets.

*Data curators* are responsible for the quality of metadata used to describe data sets, including descriptive free text as well as ontological annotations. Their needs revolve around the current state of curation and how its quality can be improved, to subsequently increase the expressiveness and findability (Morville, 2005) of data sets. *Data curators* are not concerned about retrieving specific data sets for analysis or discovering trends in data availability but instead care about the overall distribution and usage of annotation terms. Hence, *data curators* primarily need to:

**N4** get an overview of the annotation term hierarchy and term usage.

## 2.3 Tasks

To develop a better understanding of the data discovery behavior in the biomedical domain, we performed a field study with eight PhD-level scientists and one graduate PhD student from the biomedical informatics domain who had varying degrees of biomedical and computational expertise. Via semi-structured interviews we gained qualitative insights in their exploration behavior when searching for biomedical data. The results of these interviews guided our requirements analysis and design of SATORI. We identified a set of nine tasks for the three user roles that a repository exploration system needs to support.

In order to meet the needs described above, some degree of understanding of the content of a repository and its subsets is required. Subsets can either represent results of a text-based search or ontology term-based queries. Understanding the composition of attributes, i.e., annotation terms, is crucial for planning what to explore next. Hence, the requirements are separated into those that are relevant for interpreting the term composition of subsets and those that are concerned with exploring subsets of interest in the data repository.

The following five tasks are related to understanding what is contained in a collection of data sets:

**T1** Determine annotation terms of a data set.

**T2** Determine abundance of annotation terms of a collection of data sets.

**T3** Determine abundance of sets of annotation terms among a collection of data sets.

**T4** Understand annotation term containment relationships.

**T5** Summarize and view metadata of a data set.

The notion of relevant data sets (N1), i.e., data sets that significantly match an experimental attribute of interest such as *cancer*, can be achieved through the visualization of annotation terms (T1) and by viewing summaries of data set descriptions (T5). Showing the relationship between annotations terms (T4) can facilitate finding of related data sets (N2). The abundance of single ontology terms (T2) and sets of ontology terms (T3) aids the understanding of search results and can highlight trends (N3, N4). Previewing certain details of a data set (T5) can further increase or decrease relevance and help to find the desired data (N1, N2).

The following four tasks are related to the process of exploring data collections:

**T6** Search for data sets.

**T7** Query data repository by annotation terms.

**T8** Loosen annotation term constraints.

Being able to search (T6) by keywords and query (T7) by annotation terms is crucial for finding specific data sets as well as collections of data sets (N1, N2, N3, N4). Drilling down into search results by filtering according to some annotation terms (T7) or drilling up by loosening constraints (T8) supports exploration through an ontology-guided enriched search.

Finally, ranking annotations according to their abundance and size enhances both understanding the nature of data sets (N3, N4) by highlighting most abundant or most rare terms, and facilitates exploration by providing a notion of information scent (Pirolli *et al.*, 2000):

**T9** Rank annotations.

# 3 Visualization technique-dependent data processing

The treemap visualization method conveys the hierarchical order by containment; hence, data to be visualized needs to be provided in the form of a tree rather than a graph. Terms with multiple parent terms and non-terminal terms with a size greater than zero are duplicated as illustrated in Figure S1.1. The node-link diagram visualizes the hierarchy from left (the root term) to right (terminal terms). Depending on the complexity of the graph, it is possible that a node could be placed in multiple different columns, as there might be different paths to the root. To avoid visual clutter links only go in one direction: from the parent (left) to the child (right). Thus, nodes with multiple parents whose distances to their parents are not equal have to be duplicated (Figure S1.2).

# 4 Ontologies and Annotation Graphs in Neo4J

Since SATORI is integrated into the Refinery Platform, we want a relatively simple setup for storing ontologies that is space-efficient and fast in terms of subgraph retrieval. Neo4J is a graph database, which implements the property graph model. A property graph consists of nodes and edges, which can be associated to a set of properties. Neo4J additionally allows certain constraints on node and edge creation and features node labeling.

When converting an OWL ontology into the property graph model, for each satisfiable ontology class we create a node and attach a label, the uniform resource identifier (URI), and a more human-readable ontology term identifier (denoted *OntID* hereafter). The OntID is based on the Open Biomedical Ontologies (OBO) Foundry identifier. In order to be able to distinguish different types of nodes and their origin, each node representing an ontology class is labeled with *Class* and a user-defined ontology abbreviation. The ontology abbreviation is based on the *IDSPACE* of OBO's ID policy, e.g., the Experimental Factor Ontology (Malone *et al.*, 2010) is abbreviated with EFO. Labeling nodes with an ontology identifier allows to construct one large graph but still be able to trace the origin of each node. A node's URI and OntID must be unique in order to avoid ambiguities.

For each satisfiable class we extract the set of direct superclasses and relate them with a custom *subclass of* relationship. Classes with an empty set of superclasses are linked to the absolute root class (i.e., *OWL:Thing*) to ensure a unique global hierarchy of ontology classes.

In order to access the annotation graph, for each available data set a node is created with its local identifier. The node is additionally labeled with *DataSet*. For every ontology annotation the node of the data set is

related to the corresponding node of the ontology class via an *annotated with* relationship.

To enable user-specific exploration, we also create a node for each user with a local identifier and label it with *User*. The user's node is related to every data set node the user has access to.

Finally, in order to efficiently retrieve parts of the ontology graph, which account for user-specific annotation term graphs, we traverse the ontology graph in a bottom-up fashion from direct annotation terms to the absolute root term and label the nodes along the traversal. This is only done once every time a new data set is important or shared. The assigned node labels allow us to quickly fetch all user-specific annotation term-related nodes with a single query using the Cypher query language of Neo4J.

# 5 Ontology Extraction

In order to determine which ontologies are useful for exploring a certain collection of data sets, we parsed the data set descriptions, i.e., the corresponding ISA-Tab files, and extracted all unique ontology IDs. Therefore, SATORI relies on the primary data repositories imported in the Refinery Platform and does not perform additional ontology mapping. Given the extracted ontology IDs we manually search for available OWL versions of the ontologies on Bioportal (Whetzel *et al.*, 2011), Ontobee (Xiang *et al.*, 2011), AberOwl (Hoehndorf *et al.*, 2015), and the internet in general. We rely on several services to get the highest coverage of ontologies as no single platform provides sufficient access to ontology files. An overview of the imported ontologies for the two data repositories used in the evaluation (Stem Cell Commons (Ho Sui *et al.*, 2013) and MetaboLights (Haug *et al.*, 2013)) can be found at https://github.com/refinery-platform/ontology-imports.

## Supplementary Figures

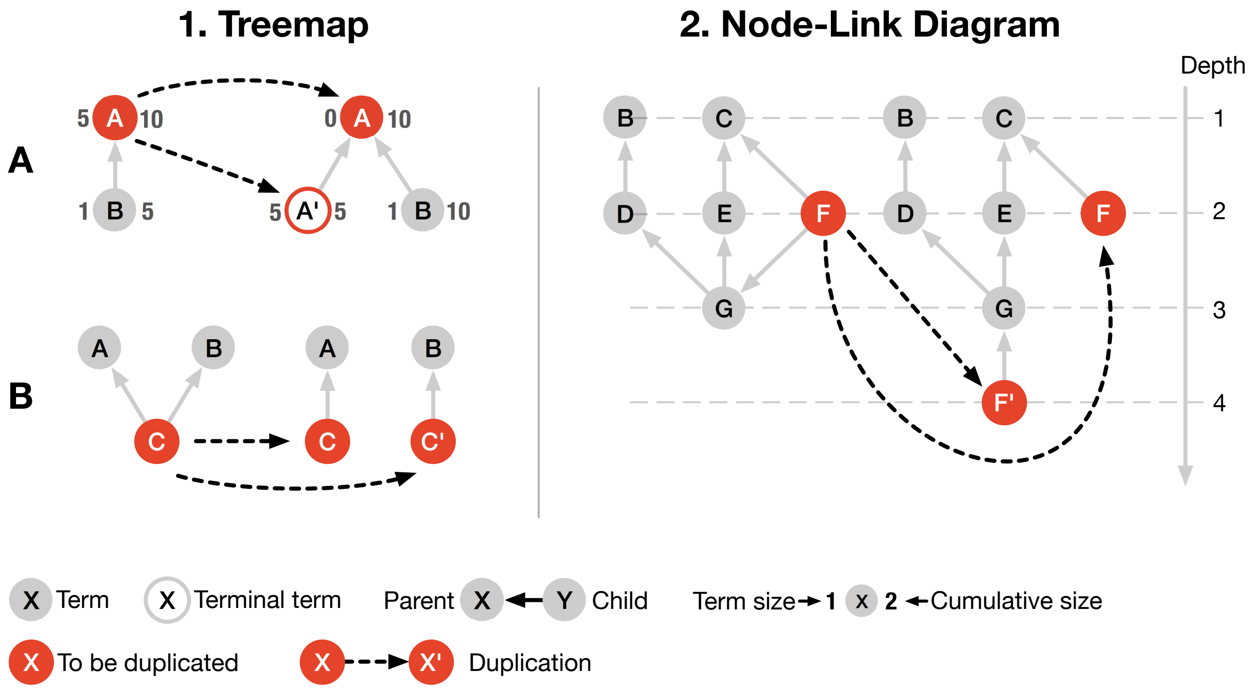


Figure S1: Node duplication for the treemap and node-link diagram. For the treemap (1), inner annotation terms with a term size (number left of a node) greater than zero need to be duplicated as child nodes to themselves (1a). Also, annotation terms with multiple parent terms are duplicated for each parent to provide a unique path to the root (1b). (2) The node-link diagram only requires annotation terms with multiple parents to be duplicated if their parents' distance to the root is not equivalent. For example, node F is duplicated because the distance of node C (1) and G (3) is not equal. On the other hand, node G is not duplicated because the distance of nodes D (2) and E (2) is the same.

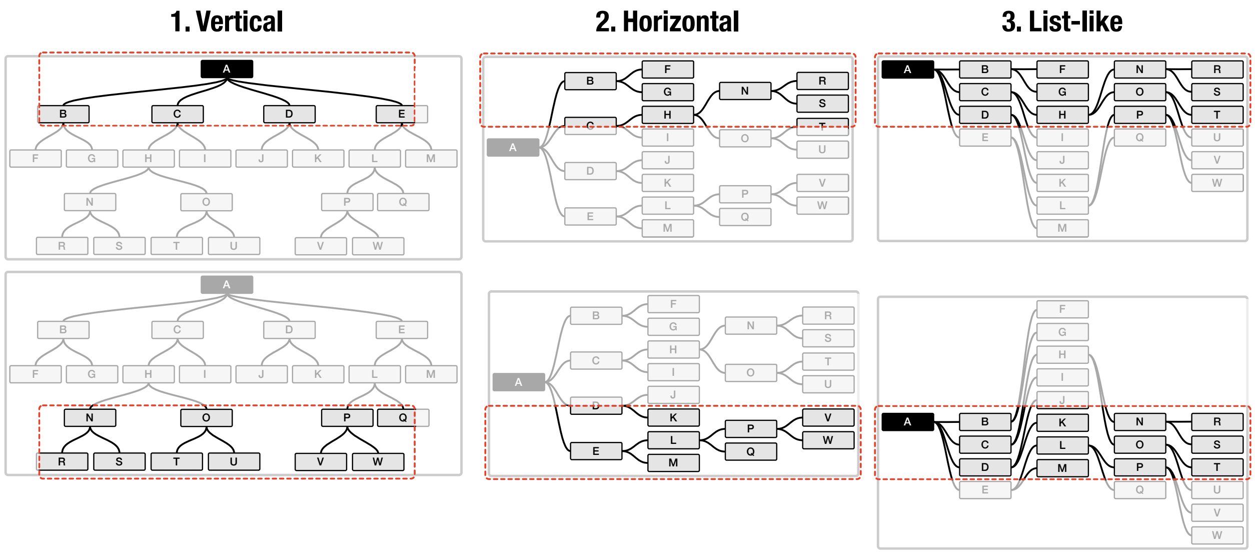


Figure S2: Comparison of the visual space efficiency between a vertical (2), horizontal (2), and list-like (3) node-link diagram. The light gray border indicates the overall space consumption and the red dotted border illustrates the available screen space. The list-like node-link diagram (3) has the most efficient space consumption. This comes at the cost of reduced legibility of the graph's structure but is especially useful when nodes are sortable. The idea is similar to the difference between precision and mean average precision in information retrieval. The position of a retrieved document does not influence the overall precision but it does have an important impact on findability. Mean average precision tries to take this into account by calculating the precision up to every position and averaging over it. Since the list-like node-link diagram in SATORI displays the precision and recall of annotation terms it is important to see the most or least abundant annotation terms first.

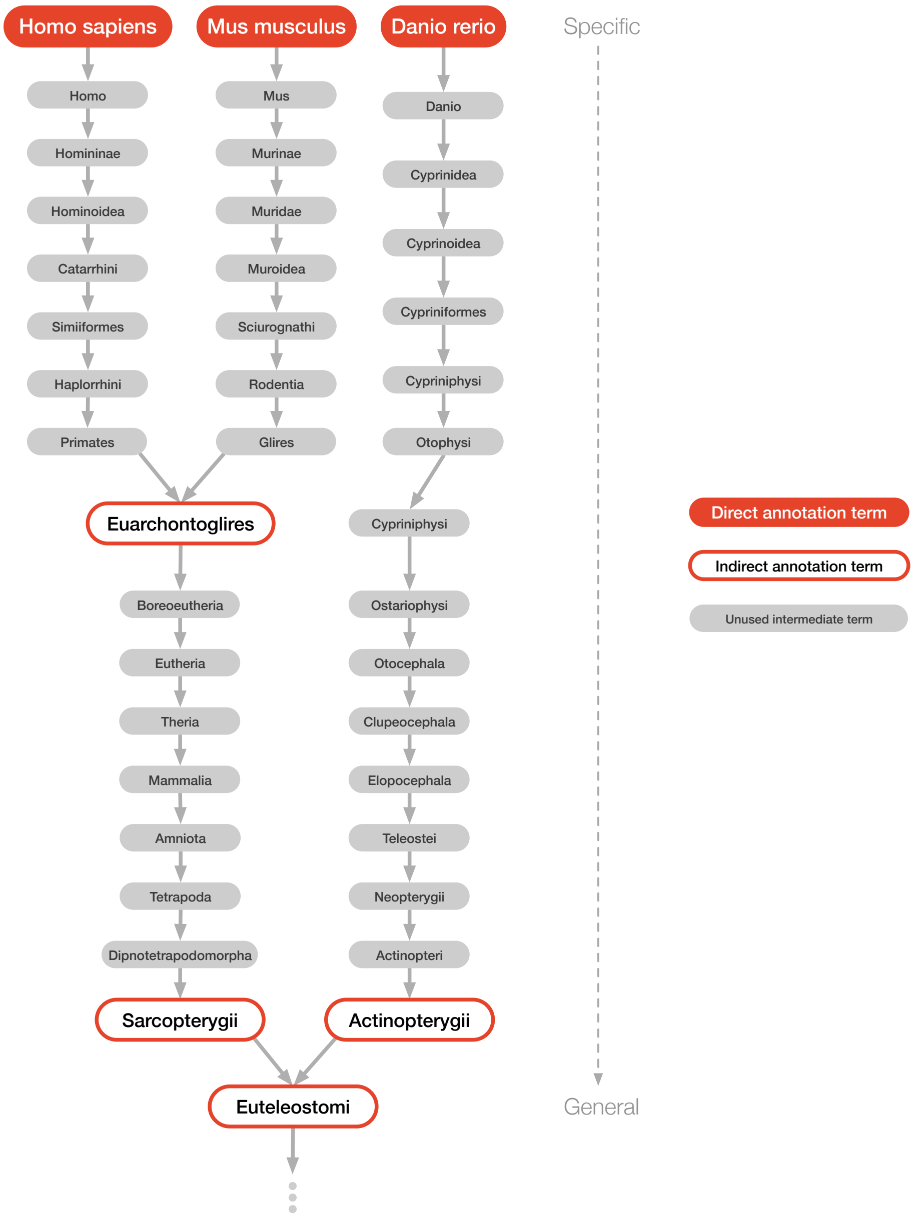


Figure S3: Pruning indirect annotation terms. Only the three terms *Homo sapiens*, *Mus musculus*, and *Danio rerio* have been used for annotations in Stem Cell Commons, i.e., are direct annotation terms. Parental terms colored with a light grey background represent the exact same set of data sets and are therefore omitted. Only the least common ancestors of the three direct annotation terms are compliant with Equation 1, and thus, are not omitted.

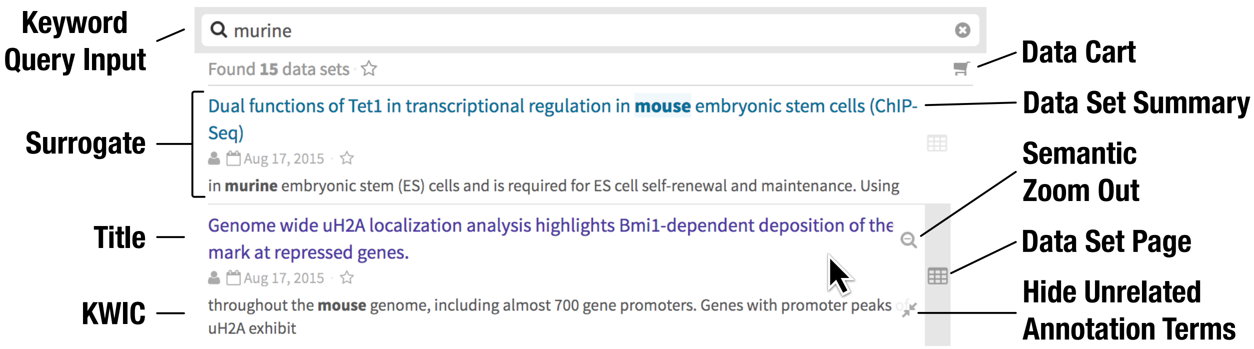


Figure S4: Search interface showing data set surrogates and buttons for exploration.

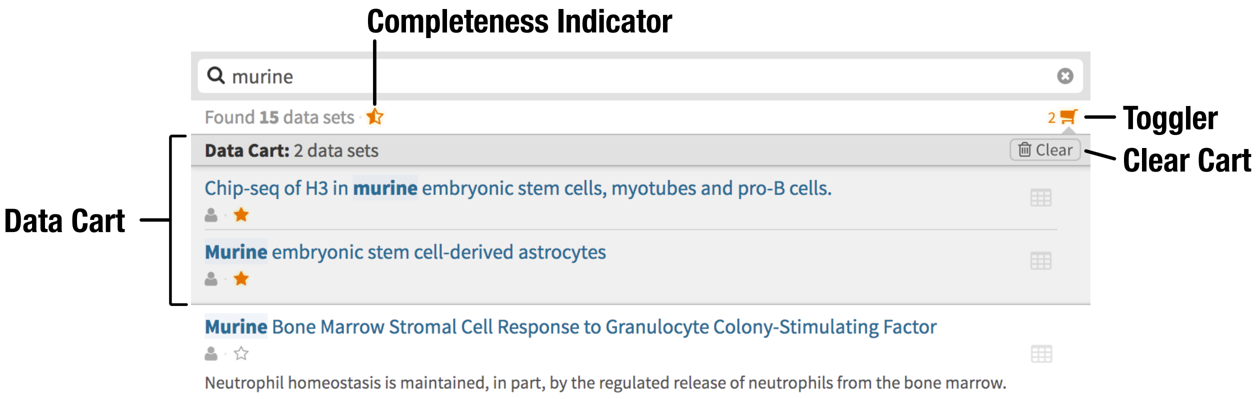


Figure S5: Data cart for temporarily collecting data sets during the exploration process. Data sets can be added and removed by clicking on the *star* icon below a data set title. Filled yellow stars represent data sets that are in the data cart. The star icon right below the search input indicates whether none, some, or all retrieved data sets have been added to the data cart. A click on the data cart icon toggles the visibility of the data cart. The data cart can be used to save interesting data sets along the exploration process. It can also be used to compare or track data sets across different searches or ontology term queries.

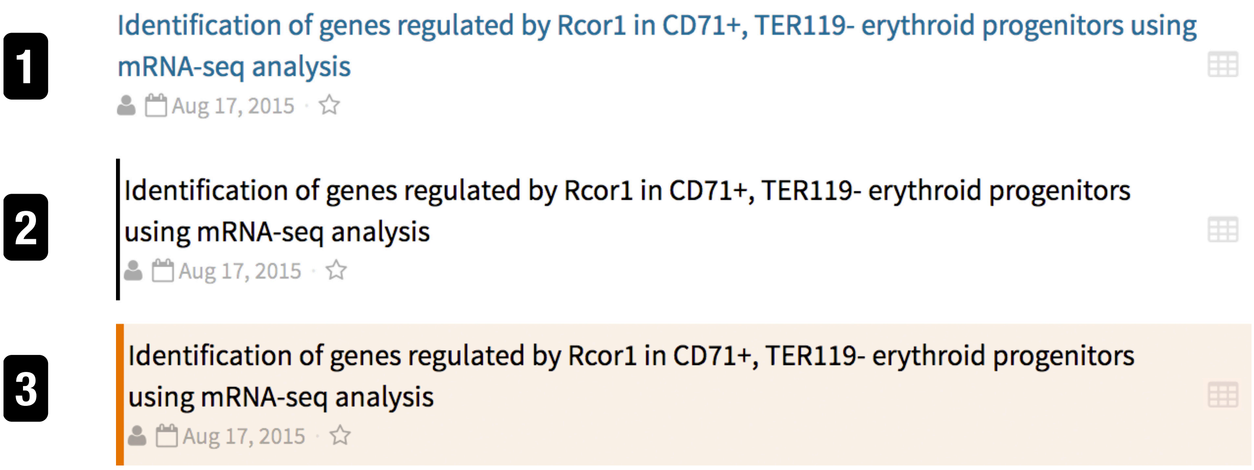


Figure S6: The three possible states of a data set surrogate in the data set list view when interacting with annotation terms: (1) normal, (2) an associated annotation term is currently hovered with the mouse cursor, and (3) an associated annotation term has been locked. Hovered (2) and locked (3) states can also be combined.

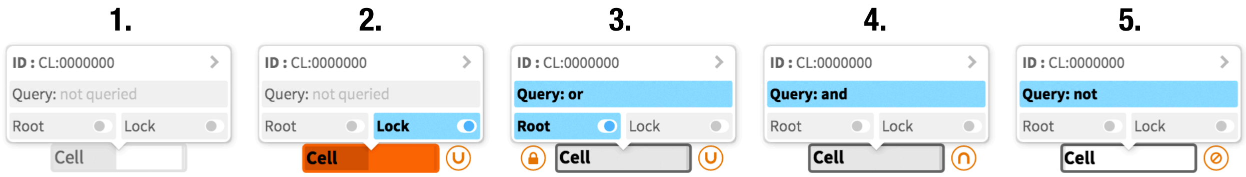


Figure S7: The annotation term context menu of the node-link diagram (1) features three interactions related to the clicked term: visually locking a term (2), re-rooting the graph by this term (3), and querying the data collection by this term (3, 4, and 5). The top most field displays the *OntID* of the term and can be toggled by a click on the arrow to reveal the complete URI. The *lock* icon to the left of a node permanently indicates when the graph is re-rooted by this term. The icon to the right of a node indicates the current query mode, i.e., *or*, *and*, or *not*. Altering the query state is achieved by clickling multiple times on the Query button.

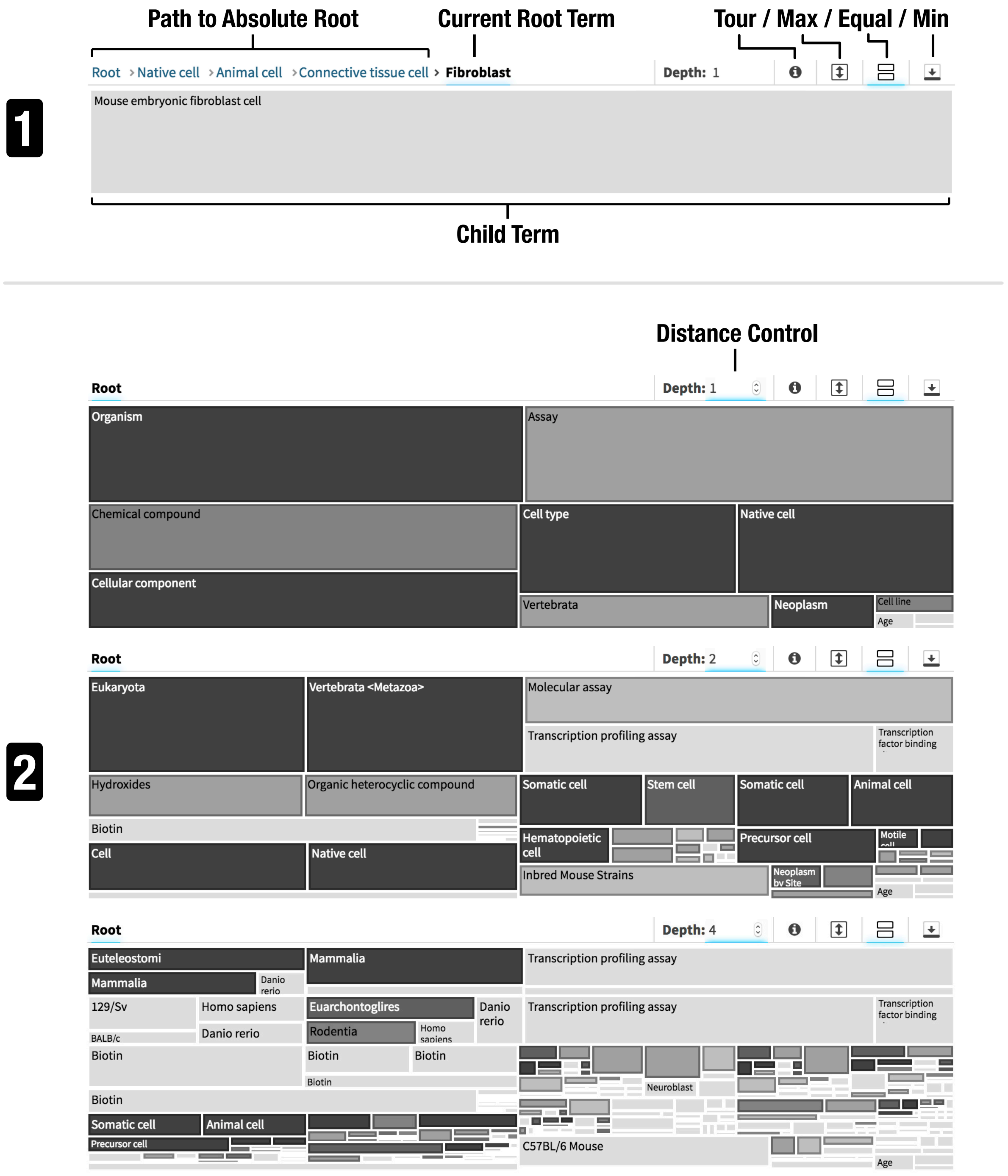


Figure S8: The breadcrumb path (1) to the absolute root term used in the treemap plot is useful for drilling up. The distance control (2) allows to view child nodes of increasing distance. The first screenshot shows all child terms of distance one to *Root*. The screenshot below shows the size distribution among inner child terms of distance two and all terminal child terms of distance one. Finally, the last screenshot illustrates inner child terms of distance four and all terminal child terms of a distance up to four. The tour button (1) starts an interactive tour for the treemap visualization. The *Max*, *Equal*, and *Min* buttons (1) set the height of the treemap to 0%, 50%, and 100% respectively.

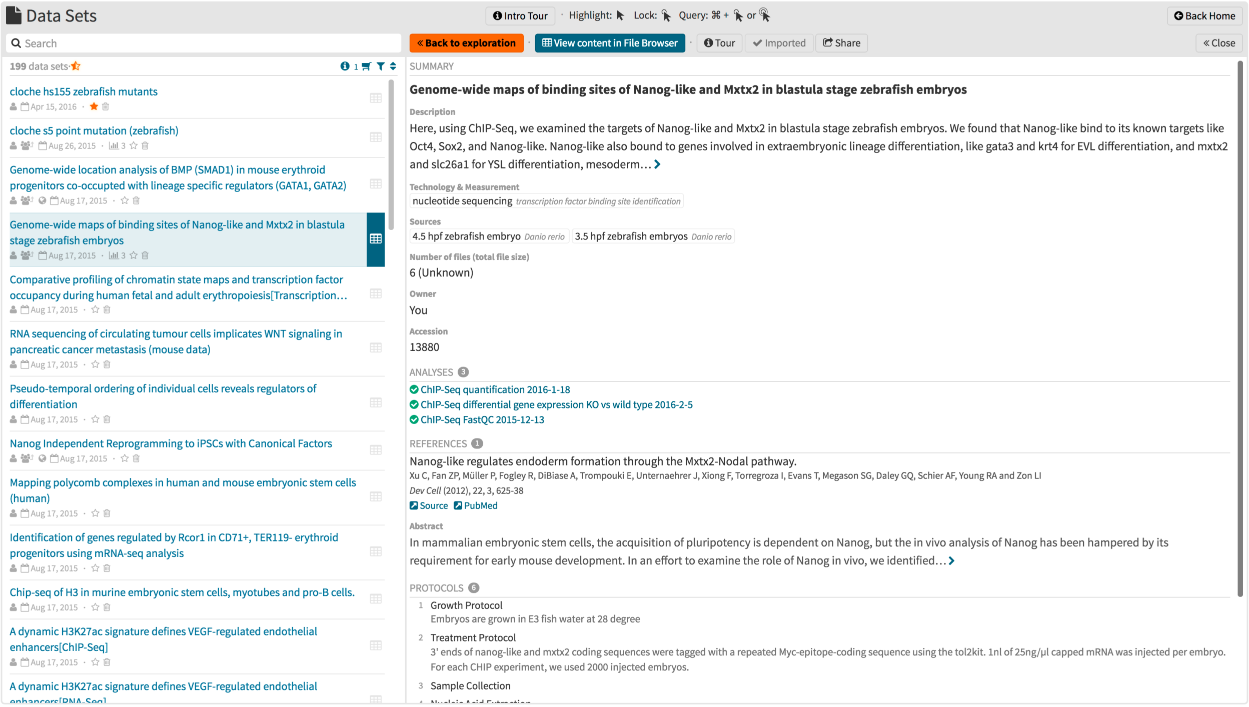


Figure S9: The data set summary view showing *Genome-wide maps of binding sites of Nanog-like and Mxtx2 in blastula stage zebrafish embryos*. The layout has been designed according to the results of question three of the initial semi-structured interview (Table S4). The first section provides a summary of the entire data set including the title, description, technology, biological sources, number of data files, ownership, and accession number of the data set. Below are all analyses listed that have been run on this data set. The next section contains related references to provide context of this data set. The final section contains the protocol used for generating the data set.

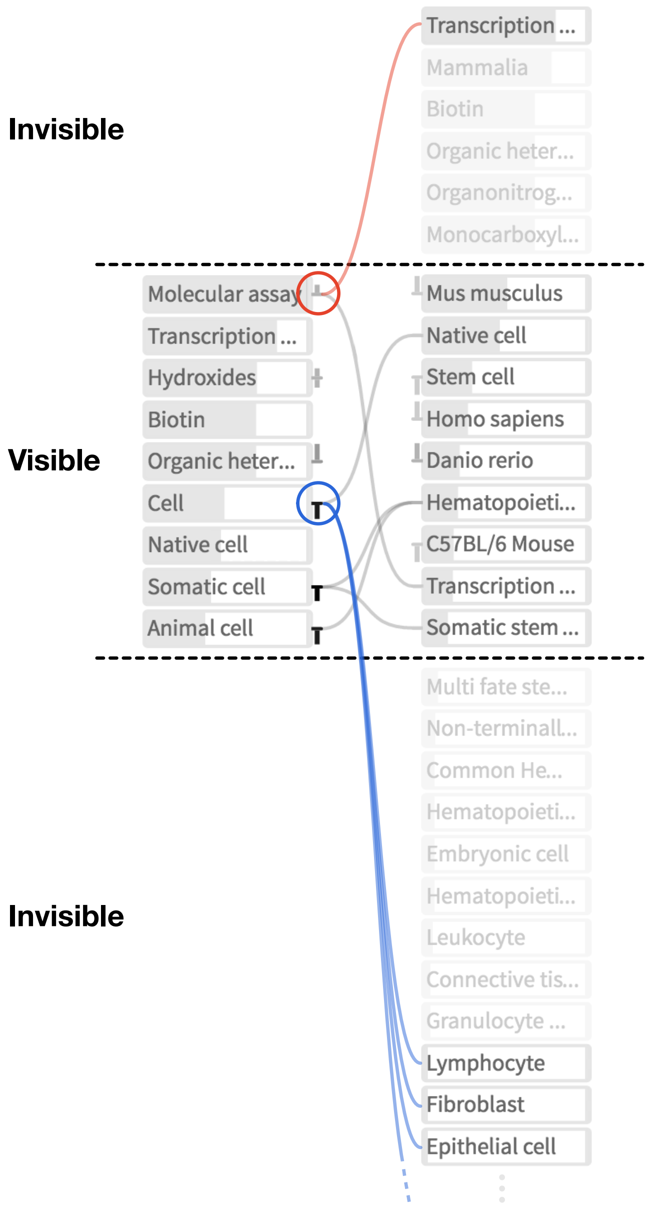


Figure S10: The link indication principle of the node-link diagram. In order to avoid clutter, only visible nodes are linked, i.e., nodes that fit within the visible area of the drawn node-link diagram, which is usually restricted by the screen size. The node link indicator bar acts as information scent (Pirolli *et al.*, 2000) by indicating where and how many linked nodes can be found in the neighboring column through scrolling. The bar's color indicates the number of related nodes that are invisible. The location (top or down) shows where the nodes in the neighboring column will appear. Finally, the height of the bar visualizes the relative distance of the farthest node that is occluded. For example, the indicator bar highlighted in red omits the red link as it points to the currently invisible node above the current scroll location of the neighboring column. The indicator bar highlighted in blue points to the bottom, is higher, and colored in a darker grey; thus, the node *cell* is linked to several nodes that are farther down in the right column as indicated by the omitted blue links. Ultimately, only the grey links are actually drawn.

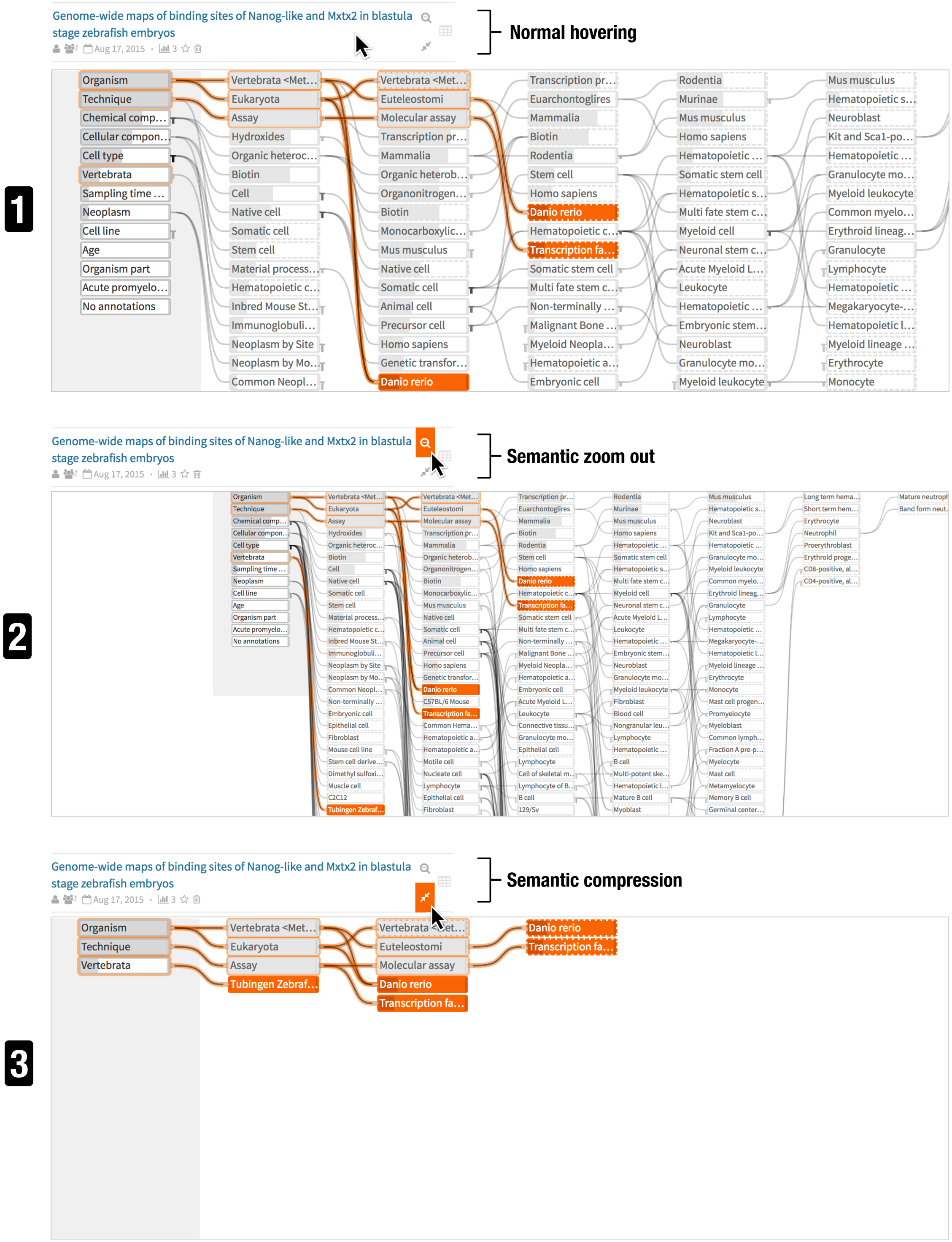


Figure S11: Highlighting data set annotations. (1) Moving the mouse cursor over a data set surrogate highlights the direct (filled in orange) and indirect (outlined in orange) annotation terms in the node link diagram. Since the graph can be larger than the available screen size, (2) semantically zooming out the annotation graph ensures that all direct annotation terms are visible. (3) The compressed annotation graph only shows the relevant annotation terms, i.e., ontology terms that are used for annotating the hovered data set.

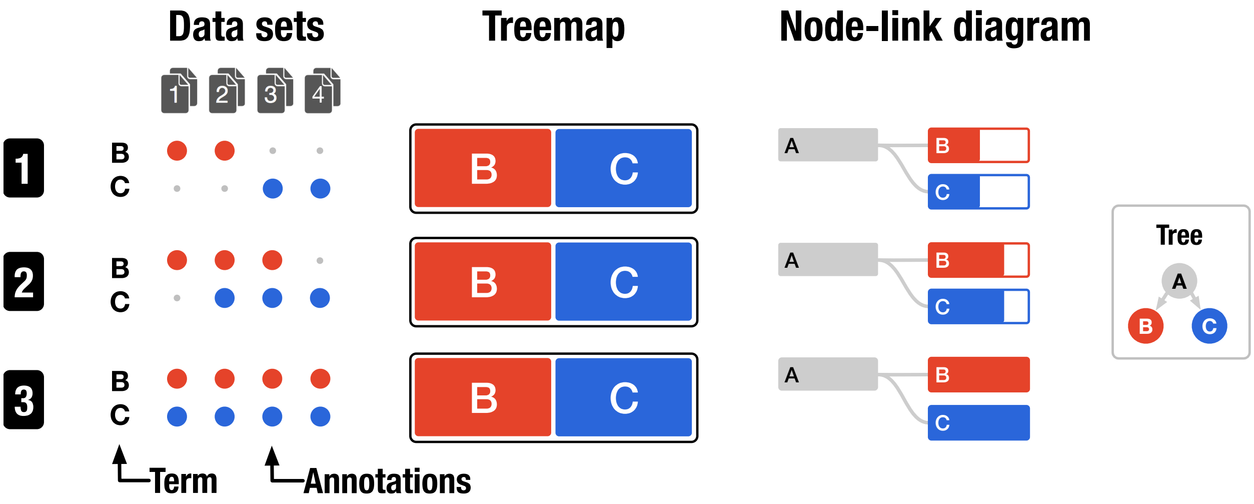


Figure S12: Difference between the representation of annotation terms in the treemap and node-link diagram. In the treemap, the area of a each rectangle is relative to other rectangles. Hence, it is not always possible to conclude how areas compare to the root “A”. E.g., the treemap looks identical in (1), (2), and (3), even though the actual size of the terms (“B” and “C”). In (1), term “B” is associated with data sets 1 and 2 and in (3) it is associated with data sets 1, 2, 3, and 4. The treemap visualization does not change as the relative size compared to term C does not change, while the node-link diagram shows the difference.

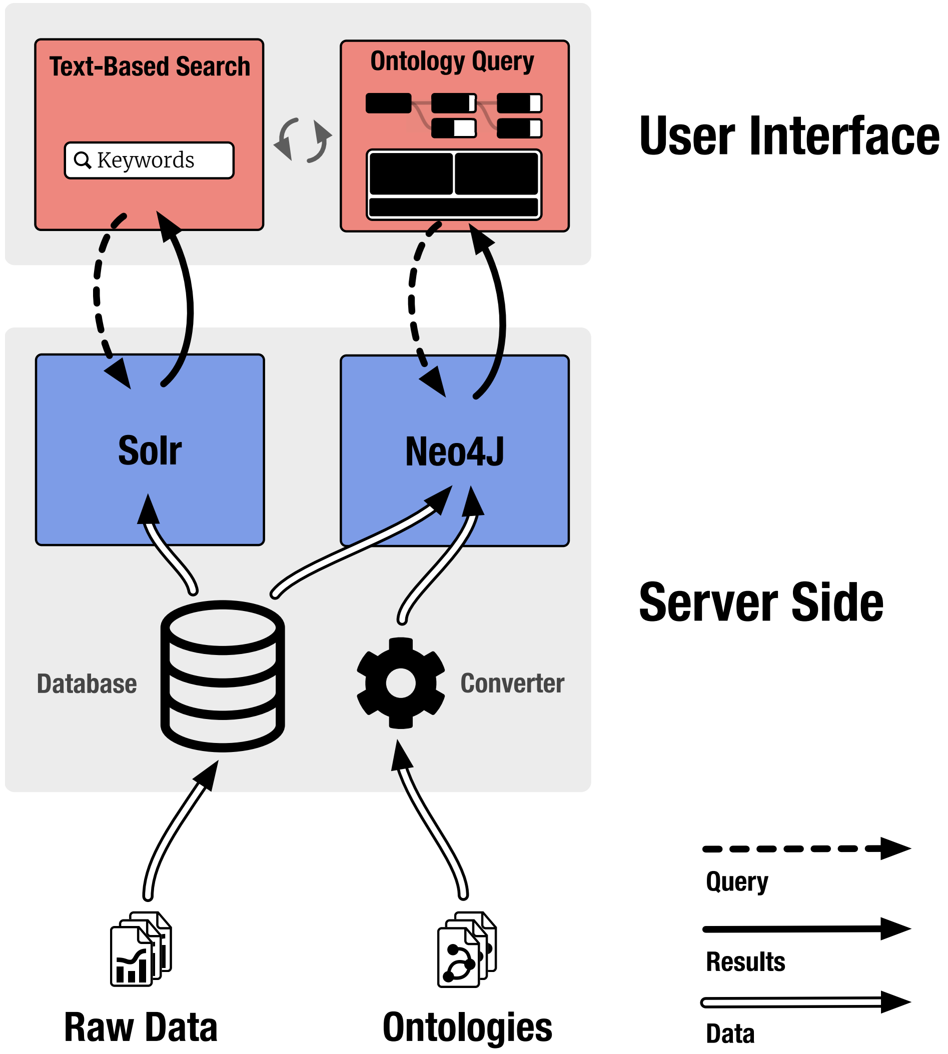


Figure S13: Overview of SATORI's system architecture. The user interface sends queries to Solr and Neo4J depending on which type of query (free text search or ontology query) is being executed. The returned results are combined on the client side. Raw data sets are imported into the Refinery Platform's database and the necessary data for the free-text search and ontology queries are further populated to Solr and Neo4J. Ontologies are converted into the property graph model (Section S4) using our custom converter, which is available at https://github.com/flekschas/owl2neo4j, and stored in Neo4J.

## Supplementary Tables

| Ontology | Number of terms | Number of data sets annotated at least once with the ontology | Number of times the ontology was used for annotation across all data sets | Number of terms used for annotation |
|---|---|---|---|---|
| BTO | 5809 | 5 | 9217 | 3 |
| CHEBI | 61550 | 144 | 22114 | 6 |
| CL | 4789 | 101 | 6020 | 59 |
| EFO | 17238 | 45 | 785 | 17 |
| FMA | 103902 | 132 | 2351 | 22 |
| GO | 44049 | 33 | 41 | 2 |
| MA | 3229 | 3 | 22 | 2 |
| NCBITAXON | 906907 | 201 | 4035 | 4 |
| NCIT | 116762 | 59 | 749 | 10 |
| OBI | 2932 | 201 | 462 | 9 |
| PATO | 2457 | 6 | 8 | 1 |
| UO | 331 | 58 | 14847 | 7 |

Table S1: Ontology usage across Stem Cell Commons data collection.

| Ontology | Number of terms | Number of data sets annotated at least once with the ontology | Number of times the ontology was used for annotation across all data sets | Number of terms used for annotation |
|---|---|---|---|---|
| AURA | 27396 | 4 | 4 | 3 |
| BAO | 6601 | 33 | 1098 | 9 |
| BioPAX | 69 | 1 | 148 | 1 |
| BTO | 5809 | 156 | 24289 | 78 |
| CARDSRVS | ? | 1 | 122 | 2 |
| CCON | 86 | 1 | 2 | 2 |
| CHEBI | 61550 | 72 | 7314 | 47 |
| CHMO | 2965 | 198 | 1698 | 32 |
| CLO | 40884 | 1 | 36 | 1 |
| CNO | 395 | 1 | 1 | 1 |
| CO-WHEAT | 176 | 1 | 1 | 1 |
| CSEO | 20085 | 17 | 11 | 2 |
| DOID | 37967 | 6 | 5 | 3 |
| DRON | 434663 | 1 | 1 | 1 |
| EDAM | 3280 | 7 | 6 | 6 |
| EFO | 17238 | 174 | 20766 | 90 |
| ENVO | 2492 | 8 | 369 | 6 |
| FIX | 1163 | 2 | 2 | 1 |
| GO | 46630 | 17 | 15 | 14 |
| HP | 15804 | 1 | 1 | 2 |
| ICF | 1596 | 1 | 1 | 1 |
| IDO | 507 | 1 | 1 | 1 |
| MEDDRA | 69107 | 2 | 59 | 1 |
| MESH | 265414 | 39 | 639 | 29 |
| MI | 1488 | 1 | 1 | 1 |
| MS | 2618 | 174 | 71620 | 54 |
| NCBITAXON | 906907 | 194 | 49045 | 98 |
| NCIT | 116762 | 128 | 5438 | 121 |
| NEMO | 1851 | 2 | 1 | 1 |
| NIFSTD | 124337 | 2 | ? | 1 |
| NMR | 481 | 2 | 2 | 1 |
| OBI | 2932 | 205 | 923 | 18 |
| PATO | 2457 | 1 | 1 | 5 |
| PO | 1972 | 10 | 473 | 7 |
| PW | 2641 | 1 | 1 | 1 |
| ROLEO | 673 | 7 | 21 | 8 |
| SIO | 1522 | 1 | 5 | 1 |
| SNOMEDCT | 327128 | 13 | 130 | 8 |
| SOY | 1835 | 1 | 1 | 1 |
| SWEET | 4550 | 2 | 4 | 3 |
| UNITSONT | 64 | 1 | 1 | 1 |
| UO | 331 | 114 | 54305 | 38 |
| XCO | 510 | 11 | 23 | 4 |

Table S2: Ontology usage across MetaboLights from a snapshot of their data collection taken in January 2017.

| | |
|---|---|
| **Q1** | Do you usually start searching at (a) a data repository such as GEO or ArrayExpress, (b) a scientific journal database such as PubMed, or (c) both depending on the context? |
| **Q1.1** | In case of Q1.b: Do you usually read the whole paper or is the abstract (plus figures) sufficient for searching? |
| **Q2** | Briefly describe how you find relevant data through the steps described in Q1. |
| **Q3** | Rate the following attributes of a data set or study by (a) importance and (b) how frequently it is important in regards to the relevance for search from 1 to 5 (1 = irrelevant ǀ never; 2 = rarely important ǀ rarely; 3 = helpful ǀ sometimes; 4 = important ǀ often; 5 = essential ǀ always): |
| **Q3.1** | Technology |
| **Q3.2** | Sample size |
| **Q3.3** | Replicates |
| **Q3.4** | Data quality |
| **Q3.5** | Species |
| **Q3.6** | Organ or tissue |
| **Q3.7** | Cell type |
| **Q3.8** | Disease |
| **Q3.9** | Cell line |
| **Q3.10** | Marker genes |
| **Q3.11** | Protocol |
| **Q3.12** | Original problem or goal |
| **Q3.13** | Publication date |
| **Q3.14** | Generating laboratory or group |
| **Q3.15** | Prominence or awareness |
| **Q3.16** | Technical popularity |
| **Q4** | Are there other attributes that are important to the relevance of a data set during search? |
| **Q5** | What are your primary purposes when searching for data? |
| **Q6** | Do you usually start your search (a) with a precise search query and loosen constraints, e.g. by reformulation when you cannot find anything relevant, or (b) with a broader search query and then specify the query? |
| **Q7** | Would it be useful to have an indicator of the number of data sets related to a certain category, which has only been found partially? E.g. a search for "liver" might not return all data sets related to "hepatic lobule" or "hepatocyte". |

Table S3: Questionnaire used for the semi-structured interviews in the field study that assesses the domain situation.

| | Q3.1 | | Q3.2 | | Q3.3 | | Q3.4 | | Q3.5 | | Q3.6 | | Q3.7 | | Q3.8 | |
|---|---|---|---|---|---|---|---|---|---|---|---|---|---|---|---|---|
| | IMP | FREQ | IMP | FREQ | IMP | FREQ | IMP | FREQ | IMP | FREQ | IMP | FREQ | IMP | FREQ | IMP | FREQ |
| Mean | 4.4 | 4.4 | 3.3 | 3.3 | 3.3 | 3.8 | 4.7 | 4.9 | 4.4 | 4.6 | 4.0 | 4.8 | 3.8 | 4.3 | 4.1 | 4.1 |
| 5 | 56% | 56% | 11% | 22% | 22% | 33% | 89% | 89% | 56% | 67% | 63% | 75% | 33% | 33% | 44% | 56% |
| 4 | 33% | 33% | 33% | 11% | 22% | 33% | 0% | 11% | 33% | 22% | 13% | 25% | 33% | 67% | 33% | 11% |
| 3 | 11% | 11% | 33% | 44% | 22% | 11% | 0% | 0% | 11% | 11% | 0% | 0% | 22% | 0% | 11% | 22% |
| 2 | 0% | 0% | 22% | 22% | 33% | 22% | 11% | 0% | 0% | 0% | 13% | 0% | 0% | 0% | 11% | 11% |
| 1 | 0% | 0% | 0% | 0% | 0% | 0% | 0% | 0% | 0% | 0% | 13% | 0% | 11% | 0% | 0% | 0% |
| 5 & 4 | 89% | 89% | 44% | 33% | 44% | 67% | 89% | 100% | 89% | 89% | 75% | 100% | 67% | 100% | 78% | 67% |
| 2 & 3 | 11% | 11% | 56% | 67% | 56% | 33% | 11% | 0% | 11% | 11% | 13% | 0% | 22% | 0% | 22% | 33% |
| SD | 0.73 | 0.73 | 1.00 | 1.12 | 1.22 | 1.20 | 1.00 | 0.33 | 0.73 | 0.73 | 1.60 | 0.46 | 1.30 | 0.50 | 1.05 | 1.17 |
| Z-score | 1.37 | 1.37 | -0.11 | -0.10 | -0.09 | 0.27 | 1.22 | 4.32 | 1.37 | 1.53 | 0.34 | 2.81 | 0.25 | 1.77 | 0.63 | 0.57 |
| Percentile | 92% | 92% | 45% | 46% | 46% | 61% | 89% | 100% | 92% | 94% | 63% | 100% | 60% | 96% | 74% | 72% |
| | **Q3.9** | | **Q3.10** | | **Q3.11** | | **Q3.12** | | **Q3.13** | | **Q3.14** | | **Q3.15** | | **Q3.16** | |
| | IMP | FREQ | IMP | FREQ | IMP | FREQ | IMP | FREQ | IMP | FREQ | IMP | FREQ | IMP | FREQ | IMP | FREQ |
| Mean | 3.3 | 4.1 | 1.9 | 4.2 | 3.0 | 4.2 | 3.0 | 3.8 | 3.0 | 3.4 | 3.0 | 4.1 | 3.2 | 4.3 | 2.7 | 4.4 |
| 5 | 11% | 22% | 0% | 56% | 0% | 44% | 11% | 44% | 22% | 14% | 0% | 33% | 0% | 44% | 0% | 56% |
| 4 | 44% | 67% | 11% | 22% | 44% | 33% | 11% | 11% | 22% | 29% | 33% | 44% | 56% | 44% | 22% | 33% |
| 3 | 22% | 11% | 11% | 11% | 22% | 22% | 44% | 22% | 11% | 43% | 33% | 22% | 11% | 11% | 33% | 11% |
| 2 | 11% | 0% | 33% | 11% | 22% | 0% | 33% | 22% | 22% | 14% | 33% | 0% | 33% | 0% | 33% | 0% |
| 1 | 11% | 0% | 44% | 0% | 11% | 0% | 0% | 0% | 22% | 0% | 0% | 0% | 0% | 0% | 11% | 0% |
| 5 & 4 | 56% | 89% | 11% | 78% | 44% | 78% | 22% | 56% | 44% | 43% | 33% | 78% | 56% | 89% | 22% | 89% |
| 2 & 3 | 33% | 11% | 44% | 22% | 44% | 22% | 78% | 44% | 33% | 57% | 67% | 22% | 44% | 11% | 67% | 11% |
| SD | 1.22 | 0.60 | 1.05 | 1.09 | 1.12 | 0.83 | 1.00 | 1.30 | 1.58 | 0.98 | 0.87 | 0.78 | 0.97 | 0.71 | 1.00 | 0.73 |
| Z-score | -0.09 | 1.10 | -1.48 | 0.71 | -0.40 | 0.93 | -0.45 | 0.25 | -0.28 | -0.02 | -0.52 | 0.85 | -0.23 | 1.25 | -0.78 | 1.37 |
| Percentile | 1.11% | 1.11% | 1.11% | 1.11% | 1.11% | 1.11% | 1.11% | 1.11% | 1.11% | 1.11% | 1.11% | 1.11% | 1.11% | 1.11% | 1.11% | 1.11% |

Table S4: Evaluation of data set attributes related to question three of Table S3. IMP stands for importance and FREQ stands for frequency. Marks 4 and 5 have been combined as an indicator for general importance and marks 2 and 3 have been combined to represent minor importance. The Z-score is based on the overall mean of importance and frequency ratings.

| | |
|---|---|
| **T1** | Find annotation terms that are associated with many data sets. |
| **T1.1** | Find terminal annotation terms that are associated with many data sets. |
| **T2** | Get an overview of native cell types. |
| **T3** | Find data sets associated with leukocyte. |
| **T4** | Get a more detailed overview of a data set's content. |
| **T5** | Compare the distribution of profiling against factor-binding data sets and evaluate whether the two collections are mutually exclusive. |

Table S5: Kickoff tasks for exploration of the Stem Cell Commons data collection, which have been used for the evaluation of SATORI.

| | | |
|---|---|---|
| BTO | tissues, cell types and enzyme sources | http://purl.obolibrary.org/obo/BTO_0000000 |
| | adult stem cell | http://purl.obolibrary.org/obo/BTO_0002666 |
| | cardiovascular system | http://purl.obolibrary.org/obo/BTO_0000088 |
| | connective tissue | http://purl.obolibrary.org/obo/BTO_0000421 |
| | embryonic structure | http://purl.obolibrary.org/obo/BTO_0000174 |
| | gland | http://purl.obolibrary.org/obo/BTO_0000522 |
| | head | http://purl.obolibrary.org/obo/BTO_0000282 |
| | hematopoietic system | http://purl.obolibrary.org/obo/BTO_0000570 |
| | immune system | http://purl.obolibrary.org/obo/BTO_0005810 |
| | integument | http://purl.obolibrary.org/obo/BTO_0000634 |
| | limb | http://purl.obolibrary.org/obo/BTO_0001492 |
| | muscular system | http://purl.obolibrary.org/obo/BTO_0001485 |
| | nervous system | http://purl.obolibrary.org/obo/BTO_0001484 |
| | respiratory system | http://purl.obolibrary.org/obo/BTO_0000203 |
| | sense organ | http://purl.obolibrary.org/obo/BTO_0000202 |
| | skeletal system | http://purl.obolibrary.org/obo/BTO_0001486 |
| | soft body part | http://purl.obolibrary.org/obo/BTO_0001262 |
| | trunk | http://purl.obolibrary.org/obo/BTO_0001493 |
| | urogenital system | http://purl.obolibrary.org/obo/BTO_0003091 |
| | viscus | http://purl.obolibrary.org/obo/BTO_0001491 |
| CHEBI | chemical entity | http://purl.obolibrary.org/obo/CHEBI_37577 |
| CL | native cell | http://purl.obolibrary.org/obo/CL_0000003 |
| EFO | measurement | http://www.ebi.ac.uk/efo/EFO_0001444 |
| | protocol | http://purl.obolibrary.org/obo/OBI_0000272 |
| | cell line | http://www.ebi.ac.uk/efo/EFO_0000322 |
| | cell type | http://www.ebi.ac.uk/efo/EFO_0000324 |
| | organism | http://purl.obolibrary.org/obo/OBI_0100026 |
| | animal component | http://www.ebi.ac.uk/efo/EFO_0000787 |
| | disease | http://www.ebi.ac.uk/efo/EFO_0000408 |
| | cell property | http://www.ebi.ac.uk/efo/EFO_0000323 |
| FMA | nucleated cell | http://purl.org/sig/ont/fma/fma67513 |
| | organ | http://purl.org/sig/ont/fma/fma67498 |
| | organ system | http://purl.org/sig/ont/fma/fma7149 |
| GO | biological process | http://purl.obolibrary.org/obo/GO_0008150 |
| | molecular function | http://purl.obolibrary.org/obo/GO_0003674 |
| MA | anatomic region | http://purl.obolibrary.org/obo/MA_0002433 |
| | organ | http://purl.obolibrary.org/obo/MA_0003001 |
| | organ system | http://purl.obolibrary.org/obo/MA_0000003 |
| | tissue | http://purl.obolibrary.org/obo/MA_0003002 |
| NCIT | neoplasm | http://ncicb.nci.nih.gov/xml/owl/EVS/Thesaurus.owl♯C3262 |
| | vertebrata | http://ncicb.nci.nih.gov/xml/owl/EVS/Thesaurus.owl♯C14282 |
| OBI | assay | http://purl.obolibrary.org/obo/OBI_0000070 |
| | organims | http://purl.obolibrary.org/obo/OBI_0100026 |
| PATO | physical object quality | http://purl.obolibrary.org/obo/PATO_0001241 |
| UO | Proteomics Standards Initiative Mass Spectrometry Vocabularies | http://purl.obolibrary.org/obo/UO_0000000 |

Table S6: Custom ontology entry point terms. Those terms have been chosen to provide meaningful entry points in regards to the exploration of data repositories given ontologies listed in Table S1.

| | | |
|---|---|---|
| BAO | assay method component | http://www.bioassayontology.org/bao♯BAO_0002753 |
| | quality | http://www.bioassayontology.org/bao♯BAO_0002928 |
| | assay biology component | http://www.bioassayontology.org/bao♯BAO_0003114 |
| BP | Gene | http://www.biopax.org/release/biopax-level3.owl♯Gene |
| | Interaction | http://www.biopax.org/release/biopax-level3.owl♯Interaction |
| | Pathway | http://www.biopax.org/release/biopax-level3.owl♯Pathway |
| | Physical Entity | http://www.biopax.org/release/biopax-level3.owl♯PhysicalEntity |
| DOID | disease | http://purl.obolibrary.org/obo/DOID_4 |
| ENVO | environmental feature | http://purl.obolibrary.org/obo/ENVO_00002297 |
| | environmental material | http://purl.obolibrary.org/obo/ENVO_00010483 |
| | environmental system | http://purl.obolibrary.org/obo/ENVO_01000254 |
| | environmental system process | http://purl.obolibrary.org/obo/ENVO_02500000 |
| ERO | technique | http://purl.obolibrary.org/obo/ERO_0000007 |
| ICF | ICF qualifier | http://who.int/icf♯ICFQualifier |
| FIX | physico-chemical method | http://purl.obolibrary.org/obo/FIX_0000096 |
| | physico-chemical property | http://purl.obolibrary.org/obo/FIX_0000268 |
| MI | interaction type | http://purl.obolibrary.org/obo/MI_0190 |
| | interactor type | http://purl.obolibrary.org/obo/MI_0313 |
| | biological role | http://purl.obolibrary.org/obo/MI_0500 |
| MS | unit | http://purl.obolibrary.org/obo/MS_0000000 |
| NCIT | Vertebrata | http://ncicb.nci.nih.gov/xml/owl/EVS/Thesaurus.owl♯C14282 |
| | Neoplasm | http://ncicb.nci.nih.gov/xml/owl/EVS/Thesaurus.owl♯C3262 |
| | Anatomic Structure, System, or Substance | http://ncicb.nci.nih.gov/xml/owl/EVS/Thesaurus.owl♯C12219 |
| | Experimental Organism Anatomical Concept | http://ncicb.nci.nih.gov/xml/owl/EVS/Thesaurus.owl♯C22188 |
| | Behavior | http://ncicb.nci.nih.gov/xml/owl/EVS/Thesaurus.owl♯C16326 |
| | Clinical or Research Activity | http://ncicb.nci.nih.gov/xml/owl/EVS/Thesaurus.owl♯C16203 |
| | Organism | http://ncicb.nci.nih.gov/xml/owl/EVS/Thesaurus.owl♯C14250 |
| | Disorder | http://ncicb.nci.nih.gov/xml/owl/EVS/Thesaurus.owl♯C7057 |
| | Drug, Food, Chemical or Biomedical Material | http://ncicb.nci.nih.gov/xml/owl/EVS/Thesaurus.owl♯C1908 |
| NEMO | ERP topography | http://purl.bioontology.org/NEMO/ontology/NEMO.owl♯NEMO_0000001 |
| PATO | process quality | http://purl.obolibrary.org/obo/PATO_0001236 |
| | physical object quality | http://purl.obolibrary.org/obo/PATO_0001241 |
| PO | plant structure development stage | http://purl.obolibrary.org/obo/PO_0009012 |
| | plant anatomical entity | http://purl.obolibrary.org/obo/PO_0025131 |
| PW | pathway | http://purl.obolibrary.org/obo/PW_0000001 |

Table S7: Additional custom ontology entry point terms for the ontologies used across MetaboLights (Table S2).

# References


Andrews, K. *et al.* (2002). The InfoSky Visual Explorer: Exploiting Hierarchical Structure and Document Similarities. *Information Visualization*, **1**(3-4), 166–181.

Andrews, K., Gutl, C., Moser, J., Sabol, V., and Lackner, W. (2001). Search result visualisation with xFIND. In *Second International Workshop on User Interfaces to Data Intensive Systems, 2001. UIDIS 2001. Proceedings*, pages 50–58.

Archambault, D., Munzner, T., and Auber, D. (2008). GrouseFlocks: Steerable Exploration of Graph Hierarchy Space. *IEEE Transactions on Visualization and Computer Graphics*, **14**(4), 900–913.

Clarkson, E. *et al.* (2009). ResultMaps: Visualization for Search Interfaces. *IEEE Transactions on Visualization and Computer Graphics*, **15**(6), 1057–1064.

Collins, C., Viegas, F., and Wattenberg, M. (2009). Parallel Tag Clouds to explore and analyze faceted text corpora. In *Proceedings of the IEEE Symposium on Visual Analytics Science and Technology (VAST '09)*, pages 91–98.

Glueck, M., Hamilton, P., Chevalier, F., Breslav, S., Khan, A., Wigdor, D., and Brudno, M. (2016). PhenoBlocks: Phenotype Comparison Visualizations. *IEEE Transactions on Visualization and Computer Graphics*, **22**(1), 101–110.

Haug, K. *et al.* (2013). MetaboLights—an open-access general-purpose repository for metabolomics studies and associated meta-data. *Nucleic Acids Research*, **41**(D1), D781–D786.

Hearst, M. (2009). *Search User Interfaces*. Cambridge University Press.

Hearst, M. A. (1995). TileBars: Visualization of Term Distribution Information in Full Text Information Access. In *Proceedings of the SIGCHI Conference on Human Factors in Computing Systems (CHI '95)*, pages 59–66. ACM.

Ho Sui, S. *et al.* (2013). The Stem Cell Commons: an exemplar for data integration in the biomedical domain driven by the ISA framework. *AMIA Joint Summits on Translational Science proceedings AMIA Summit on Translational Science*, **2013**, 70.

Hoeber, O. and Xue Dong Yang (2006). The Visual Exploration ofWeb Search Results Using HotMap. In *Information Visualization 2006 (IV 2006)*, pages 157–165. IEEE.

Hoehndorf, R., Slater, L., Schofield, P. N., and Gkoutos, G. V. (2015). Aber-owl: a framework for ontology-based data access in biology. *BMC bioinformatics*, **16**(1), 26.

Johnson, B. and Shneiderman, B. (1991). Tree-maps: a space-filling approach to the visualization of hierarchical information structures. In *Proceedings of the IEEE Conference on Visualization (Vis '91)*, pages 284–291.

Katifori, A., Halatsis, C., Lepouras, G., Vassilakis, C., and Giannopoulou, E. (2007). Ontology visualization methods—a survey. *ACM Computing Surveys*, **39**(4), 10–es.

Lohmann, S., Negru, S., Haag, F., and Ertl, T. (2014). VOWL 2: User-Oriented Visualization of Ontologies. In K. Janowicz, S. Schlobach, P. Lambrix, and E. Hyvönen, editors, *Knowledge Engineering and Knowledge Management*, volume 8876, pages 266–281. Springer International Publishing, Cham.

Malone, J., Holloway, E., Adamusiak, T., Kapushesky, M., Zheng, J., Kolesnikov, N., Zhukova, A., Brazma, A., and Parkinson, H. (2010). Modeling sample variables with an experimental factor ontology. *Bioinformatics*, **26**(8), 1112–1118.

Morville, P. (2005). *Ambient Findability: What We Find Changes Who We Become*. "O'Reilly Media, Inc.".

Pirolli, P. *et al.* (2000). The Effect of Information Scent on Searching Information: Visualizations of Large Tree Structures. In *Proceedings of the Working Conference on Advanced Visual Interfaces*, AVI '00, pages 161–172, New York, NY, USA. ACM.

Reiterer, H., Tullius, G., and Mann, T. M. (2005). INSYDER: a content-based visual-information-seeking system for the Web. *International Journal on Digital Libraries*, **5**(1), 25–41.

Robinson, P. N., Köhler, S., Bauer, S., Seelow, D., Horn, D., and Mundlos, S. (2008). The Human Phenotype Ontology: A Tool for Annotating and Analyzing Human Hereditary Disease. *The American Journal of Human Genetics*, **83**(5), 610–615.

Schulz, H.-J. (2011). Treevis.net: A Tree Visualization Reference. *IEEE Computer Graphics and Applications*, **31**(6), 11–15.

Smith, A., Hawes, T., and Myers, M. (2014). Hierarchie: Visualization for Hierarchical Topic Models. In *Proceedings of the Workshop on Interactive Language Learning, Visualization, and Interfaces*, pages 71–78. Association for Computational Linguistics.

Stasko, J., Görg, C., and Liu, Z. (2007). Jigsaw: Supporting Investigative Analysis through Interactive Visualization. In *Proceedings of the IEEE Symposium on Visual Analytics in Science and Technology*, VAST '07, pages 131–138. IEEE.

Whetzel, P. L., Noy, N. F., Shah, N. H., Alexander, P. R., Nyulas, C., Tudorache, T., and Musen, M. A. (2011). Bioportal: enhanced functionality via new web services from the national center for biomedical ontology to access and use ontologies in software applications. *Nucleic acids research*, **39**(suppl_2), W541–W545.

Xiang, Z., Mungall, C., Ruttenberg, A., and He, Y. (2011). Ontobee: A linked data server and browser for ontology terms. In *ICBO*.

Zhang, J. and Marchionini, G. (2004). Coupling browse and search in highly interactive user interfaces: a study of the relation browser++. In *Proceedings of the 4th ACM/IEEE-CS joint conference on Digital libraries*, page 384. ACM Press.

Zhao, S., McGuffin, M., and Chignell, M. (2005). Elastic hierarchies: combining treemaps and node-link diagrams. In *Proceedings of the IEEE Symposium on Information Visualization (InfoVis '05)*, pages 57–64. IEEE Computer Society Press.
